# Supplementary material for: Landform and lithospheric development contribute to the assembly of mountain floras in China
Source: Nat Commun. 2024 Jun 17;15:5139. doi: 10.1038/s41467-024-49522-4 (PMC11183111; doi:10.1038/s41467-024-49522-4)
Supplement: Supplementary file 1 — Supplementary Information [file 41467_2024_49522_MOESM1_ESM.pdf]

Supplementary Information:

Supplementary Table 1 The checklists of 140 mountain floras used in this study.

Num.	Procince	Mountain flora	Acronym	Tectonic type	Landform type	Area (km^2)	Longitude (E)	Latitude (N)
1	Jiangxi	Xiaowudang[1]	jxxwd	orogenic	Danxia	13.5	114.721660	24.612923
2	Guangdong	Danxiashan[2]	gddxs	orogenic	Danxia	292.0	113.738866	25.015979
3	Guangdong	Nanxiongdanxia[3]	gdnxdx	orogenic	Danxia	23.6	114.196079	25.140916
4	Jiangxi	Hanxianyan[1]	jxhxy	orogenic	Danxia	75.0	115.808300	25.200044
5	Jiangxi	Luohanyan[1]	jxlhy	orogenic	Danxia	10.0	116.168703	25.999337
6	Hunan	Langshang[4]	hnlangs	craton	Danxia	108.0	110.806533	26.396407
7	Fujian	Tainingdanxia[5]	fjtndx	orogenic	Danxia	234.9	117.074066	26.890974
8	Jiangxi	Longhushan[1]	jxlhs	orogenic	Danxia	220.0	116.975421	28.095946
9	Sichuan	Huagaoxi[6]	schgx	craton	Danxia	238.3	105.537794	28.275538
10	Jiangxi	Guifeng[1]	jxgf	orogenic	Danxia	136.0	117.372524	28.320805
11	Qinghai	Sjiangyuan[7]	qhsjy	craton	Desert	395000.0	97.261475	33.024621
12	Qinghai	Huangheyuan[8]	qhhhy	orogenic	Desert	436.0	97.424011	34.794634
13	Xinjiang	Xikunlunshan[9]	xjxkls	orogenic	Desert	300000.0	79.538962	36.165253
14	Gansu	Qilianshan[10]	gsqls	orogenic	Desert	2062.0	101.610756	37.640065
15	Neimenggu	Helanshan[11]	nmghls	craton	Desert	885.0	105.964396	38.873439
16	Xinjiang	Luobupo[12]	xjlbp	craton	Desert	78000.0	89.954063	39.588288
17	Neimenggu	Qilaotushan[13]	nmgqlts	craton	Desert	11000.0	117.795695	42.615351
18	Xinjiang	Xitianshan[14]	xjxts	orogenic	Desert	27700.0	85.163193	43.674311
19	Xinjiang	Dongtianshan[15]	xjdts	orogenic	Desert	26500.0	88.456227	43.828050
20	Neimenggu	Alukeerqin[16]	nmgalkeq	orogenic	Desert	1367.9	119.931700	43.895877
21	Xinjiang	Bozhounanbu[17]	xjblnb	orogenic	Desert	5093.3	84.298096	44.107310
22	Neimenggu	Saihanwula[18]	nmgshwl	orogenic	Desert	1004.0	118.647924	44.251478
23	Xinjiang	Kelamayi[19]	xjklmy	orogenic	Desert	9500.0	84.789884	45.578491
24	Hainan	Houmiling[20]	hnhml	orogenic	Karst_Gr	122.2	109.091148	18.913107
25	Guangxi	Cenwanglaoshan[21]	gxcwls	craton	Karst_Gr	189.9	106.404344	24.499899
26	Guangxi	Yachang[22]	gxyc	craton	Karst_Gr	220.6	106.338511	24.782709
27	Guizhou	Nayong[23]	gzny	craton	Karst_Gr	114.0	105.341967	26.762852
28	Guizhou	Zhujiashan[24]	gzzjs	craton	Karst_Gr	76.4	107.639239	26.983206
29	Yunnan	Yaoshan[25]	ynys	craton	Karst_Gr	201.4	103.044380	27.237795
30	Guizhou	Fodingshan[26]	gzfds	craton	Karst_Gr	152.0	108.108273	27.337433
31	Yunnan	Zhaotongbeibu[27]	ynztbb	craton	Karst_Gr	450.0	103.877887	27.631134
32	Guizhou	Yinjiangyangxi[28]	gzyjyx	craton	Karst_Gr	218.7	108.513666	27.691466
33	Hunan	Wulingyuan[29]	hnwly	craton	Karst_Gr	369.0	110.431569	29.380863
34	Sichuan	Emeishan[30]	scems	craton	Karst_Gr	154.0	103.327029	29.519983
35	Hubei	Houhe[31]	hbhh	craton	Karst_Gr	103.4	110.559540	30.078601
36	Hubei	Shennongjia[32]	hbsnj	craton	Karst_Gr	12837.0	110.279186	31.490496
37	Shanxi	Wulushan[33]	sxwls	craton	Granitic	206.2	111.189177	36.581741
38	Hainan	Diaoluoshan[34]	hndls	orogenic	Granitic	183.9	109.863383	18.747009
39	Hainan	Jianfengling[35]	hnjfl	orogenic	Granitic	200.0	108.881158	18.755486
40	Hainan	Wuzhishan[36]	hnwzs	orogenic	Granitic	80.0	109.706696	18.913251

Num.	Procince	Mountain flora	Acronym	Tectonic type	Landform type	Area (km^2)	Longitude (E)	Latitude (N)
41	Hainan	Yinggeling[37]	hnygl	orogenic	Granitic	504.6	109.525487	19.045745
42	Hainan	Bawangling[38]	hnbwl	orogenic	Granitic	299.8	109.196929	19.087155
43	Guangxi	Shiwandashan[39]	gxswds	orogenic	Granitic	616.9	107.957454	21.893650
44	Guangdong	Ehuangzhang[40]	gdehz	orogenic	Granitic	144.5	111.457604	21.893709
45	Guangdong	Yunkaishan[41]	gdyks	orogenic	Granitic	125.1	111.198090	22.275547
46	Guangdong	Dinghushan[42]	gddhs	orogenic	Granitic	11.6	112.515233	23.182765
47	Guangdong	Xiangtoushan[43]	gdxts	orogenic	Granitic	107.0	114.395985	23.306850
48	Guangdong	Heishiding[44]	gdhsd	orogenic	Granitic	42.0	111.869393	23.463809
49	Guangxi	Damingshan[45]	gxdms	craton	Granitic	169.9	108.432321	23.478308
50	Taiwan	Taiwanshanmai[46]	twsm	orogenic	Granitic	36000.0	121.113281	23.563987
51	Yunnan	Yuanjiang[47]	ynyj	craton	Granitic	466.7	101.955931	23.617135
52	Guangdong	Nankunshan[48]	gdnks	orogenic	Granitic	18.9	113.840087	23.632136
53	Yunnan	Lancangjiang[49]	ynlcj	craton	Granitic	751.9	99.162598	23.995035
54	Yunnan	Yizushan[50]	ynyzs	craton	Granitic	1107.2	101.675149	23.999811
55	Guangxi	Daguishan[51]	gxdgs	orogenic	Granitic	37.8	111.718467	24.156766
56	Guangxi	Dayaoshan[52]	gxdys	orogenic	Granitic	256.0	110.246070	24.182143
57	Guangdong	Shimentai[53]	gdsmt	orogenic	Granitic	335.6	113.304768	24.434960
58	Jiangxi	Jiulianshan[54]	jxjlians	orogenic	Granitic	134.1	114.540122	24.662487
59	Guangxi	Xilingshan[55]	gxxls	orogenic	Granitic	193.3	111.214892	24.765648
60	Guangdong	Nanling[56]	gdnl	orogenic	Granitic	587.0	112.939453	24.839088
61	Hunan	Lanshan[57]	hnlans	orogenic	Granitic	70.5	112.011795	25.070673
62	Guangxi	Jiuwanshan[58]	gxjws	craton	Granitic	252.1	108.755739	25.182968
63	Fujian	Liangyeshan[59]	fjlys	orogenic	Granitic	143.7	116.127130	25.184593
64	Guangxi	Sanpihu[60]	gxsph	craton	Granitic	31.1	107.167474	25.320134
65	Guangxi	Yuanbaoshan[61]	gxybs	craton	Granitic	146.7	109.167022	25.397376
66	Hunan	Dupangling[62]	hndpl	craton	Granitic	200.7	111.317758	25.462202
67	Hunan	Daguping[63]	hndgp	craton	Granitic	134.7	111.617952	25.729162
68	Jiangxi	Qiyunshan[64]	jxqys	orogenic	Granitic	171.1	114.005382	25.890078
69	Hunan	Bamianshan[65]	hnbms	orogenic	Granitic	109.7	113.654435	25.926073
70	Guizhou	Yueliangshan[66]	gzyls	craton	Granitic	345.6	108.274069	25.944528
71	Jiangxi	Ganjiangyuan[67]	jxgjy	orogenic	Granitic	161.0	116.370548	26.042802
72	Hunan	Jintongshan[68]	hnjts	craton	Granitic	184.7	110.204369	26.156223
73	Guangxi	Yinzhulaoshan[69]	gxyzls	craton	Granitic	54.7	110.578151	26.281733
74	Jiangxi	Nanfegnmian[70]	jxnfm	orogenic	Granitic	105.9	114.039910	26.313425
75	Fujian	Junzifeng[71]	fjjzf	orogenic	Granitic	138.5	117.208096	26.337936
76	Guizhou	Leigongshan[72]	gzlgs	craton	Granitic	477.9	108.216687	26.382360
77	Hunan	Shunhuangshan[73]	hnshs	craton	Granitic	131.4	111.043147	26.401115
78	Hunan	Taoyuandong[74]	hntyd	orogenic	Granitic	237.9	114.010819	26.459181
79	Guizhou	Nangong[75]	gzng	craton	Granitic	221.0	108.295864	26.615687
80	Jiangxi	Jinggangshan[76]	jxjgs	orogenic	Granitic	708.7	114.146053	26.637238
81	Jiangxi	Qixiling[77]	jxqxl	orogenic	Granitic	105.0	114.195618	26.754804
82	Fujian	Minjiangyuan[78]	fjmjy	orogenic	Granitic	130.2	116.893291	26.776772

Num.	Procince	Mountain flora	Acronym	Tectonic type	Landform type	Area (km^2)	Longitude (E)	Latitude (N)
83	Fujian	Longqishan[79]	fjlqs	orogenic	Granitic	156.9	117.506037	26.883812
84	Fujian	Donggongshanbeiduan[80]	fjdgsbd	orogenic	Granitic	10500.0	119.137078	27.112386
85	Jiangxi	Gaotianyan[81]	jxgty	orogenic	Granitic	47.8	114.016113	27.373901
86	Jiangxi	Wugongshan[82]	jxwgs	orogenic	Granitic	170.0	114.172842	27.463325
87	Jiangxi	Dagangshan[83]	jxdgs	orogenic	Granitic	12.0	114.530271	27.604151
88	Yunnan	Dulongjiangliuyu[84]	yndljly	orogenic	Granitic	1947.0	98.349127	27.725759
89	Fujian	Wuyishan[85]	fjwys	orogenic	Granitic	565.3	117.719879	27.746746
90	Jiangxi	Matoushan[86]	jxmts	orogenic	Granitic	138.7	117.245081	27.796775
91	Jiangxi	Yangjifeng[87]	jxyjf	orogenic	Granitic	109.5	117.344803	27.919907
92	Jiangxi	Wuyishan[88]	jxwys	orogenic	Granitic	160.1	117.705870	28.307835
93	Hunan	Daweishan[89]	hndws	craton	Granitic	62.9	113.800484	28.485315
94	Jiangxi	Guanshan[90]	jxgs	craton	Granitic	115.0	114.552236	28.542096
95	Hunan	Xiaoxi[91]	hnxx	craton	Granitic	248.0	110.194214	28.812598
96	Jiangxi	Sanqingshan[92]	jxsqs	craton	Granitic	756.6	118.055159	28.910956
97	Hunan	Mufushan[93]	hnmfs	craton	Granitic	85.3	113.829362	28.970395
98	Jiangxi	Jiulingshan[94]	jxjlings	craton	Granitic	115.4	115.175369	29.079383
99	Jiangxi	Yishan[95]	jxys	craton	Granitic	115.1	115.025055	29.371792
100	Hubei	Jiugongshan[96]	hbjgs	craton	Granitic	166.1	114.603388	29.376972
101	Jiangxi	Lushan[97]	jxls	craton	Granitic	292.3	115.977656	29.545054
102	Chongqing	Jinyunshan[98]	cjjys	craton	Granitic	76.0	106.384231	29.848558
103	Anhui	Qingliangfeng[99]	ahqlf	craton	Granitic	78.1	118.862287	30.101334
104	Anhui	Huangshan[100]	ahhs	craton	Granitic	160.0	118.156407	30.129679
105	Zhejiang	Tianmushan[101]	zjtms	craton	Granitic	43.0	119.409571	30.353817
106	Anhui	Dabieshan[102]	dbs	orogenic	Granitic	15293.0	115.774113	31.257177
107	Chongqing	Yintiaoling[103]	cqytl	craton	karst-gr	224.2	109.931515	31.467145
108	Sichuan	Tangjiahe[104]	sctjh	orogenic	Granitic	400.0	104.755738	32.589005
109	Henan	Gaoleshan[105]	hngls	orogenic	Granitic	90.6	113.618626	32.661778
110	Shannxi	Motianling[106]	sxmtl	orogenic	Granitic	85.2	108.034842	32.731580
111	Gansu	Yuhe[107]	gsyh	orogenic	Granitic	510.6	105.442238	32.995141
112	Shannxi	Xinkailing[108]	sxxkl	orogenic	Granitic	149.9	110.698440	33.366687
113	Shannxi	Pingheliang[109]	sxphl	orogenic	Granitic	211.5	108.367164	33.400144
114	Shannxi	Changqingshan[110]	sxcqs	orogenic	Granitic	300.0	107.507497	33.618564
115	Shannxi	Taibaishan[111]	sxtbs	orogenic	Granitic	563.3	107.561869	33.930257
116	Shandong	Taishan[112]	sdts	craton	Granitic	242.0	117.083812	36.263112
117	Hebei	Tuoliang[113]	hbtl	craton	Granitic	213.1	113.811522	38.724818
118	Beijing	Yunmengshan[114]	bjyms	craton	Granitic	22.1	116.812576	40.545743
119	Liaoning	Baishilizi[115]	lnbslz	craton	Granitic	74.7	124.876008	40.926903
120	Hebei	Maojingba[116]	hbmjb	craton	Granitic	400.4	118.167158	41.676071
121	Hainan	Wangxia[117]	hnwxshy	orogenic	Karst	10.0	109.046215	19.068908
122	Guangxi	Nonggang[118]	gxng	craton	Karst	100.8	106.944879	22.485805
123	Guangxi	Longhushan[119]	gxlhs	craton	Karst	22.6	107.628250	23.012756

Num.	Procince	Mountain flora	Acronym	Tectonic type	Landform type	Area (km^2)	Longitude (E)	Latitude (N)
124	Guangxi	Laohutiao[120]	gxlht	craton	Karst	270.1	105.710269	23.100987
125	Guizhou	Xingyipogang[121]	gzxypg	craton	Karst	167.6	105.098666	25.112122
126	Guangxi	Mulun[122]	gxml	craton	Karst	90.0	108.054318	25.147779
127	Guizhou	Wangmo[123]	gzwm	craton	Karst	300.0	106.367952	25.234082
128	Guizhou	Maolan[124]	gzml	craton	Karst	212.9	107.925745	25.246513
129	Guizhou	Badashan[125]	gzbds	craton	Karst	260.0	104.835215	25.996729
130	Guizhou	Meitanbaimianshui[126]	gzmtbms	craton	Karst	185.0	107.375311	27.529538
131	Guizhou	Sinansiyetun[127]	gzsnsyt	craton	Karst	174.0	107.990337	27.896504
132	Guizhou	Kuankuoshui[128]	gzkks	craton	Karst	262.3	107.195465	28.243703
133	Hunan	Dehang[129]	hndh	craton	Karst	108.5	109.587340	28.344720
134	Guizhou	Mayanghe[130]	gzmyh	craton	Karst	311.1	108.109589	28.623104
135	Chongqing	Jinfoshan[131]	cqjfs	craton	Karst	418.5	107.181002	29.043814
136	Guizhou	Dashahe[132]	gzdsh	craton	Karst	269.9	107.603336	29.155062
137	Hunan	Wulongshan[133]	hnwls	craton	Karst	402.0	109.326685	29.210095
138	Hubei	Qizimeishan[134]	hbqzms	craton	Karst	345.5	109.746612	30.027216
139	Hubei	Jingshanyumai[135]	hbjsym	craton	Karst	2231.0	112.221616	30.998712
140	Guangdong	Qiniangshan[136,137]	gdqns	orogenic	Granitic	46.1	114.545361	22.522300

Supplementary Table 2 Predictor variables and explanatory variables.

Variables	Meaning of each variable
**Predictor variables**	
sp.number	species richness, the total number of angiosperm plants in a mountain
NRI	standardized effect size of mean phylogenetic distance
NTI	standardized effect size of mean nearest taxon distance
PDI	standardized effect size of phylogenetic diversity (PD_{Faith})
MDT	mean diversity time of species in a mountain
MDT.oldest	mean diversity time of 25% oldest species in a mountain
MDT.youngest	mean diversity time of 25% youngest species in a mountain
**Explanatory variables**	
Landform	the landform type of a mountain (included 5 variable, see method)
Tectonic	the tectonic zone which a mountain located (craton and orogenic)
Geographic (included the following 5 variable)	
area	mountain area of hectares
lat	latitude
long	longitude
elevdiff	difference value between the highest elevation and lowest elevation
elevmid	average of highest elevation and lowest elevation of a mountain
Climate (included the following 19 variable)	
bio1	Annual Mean Temperature [excluded, *r* with bio11 = 0.9609]
bio2	Mean Diurnal Range
bio3	Isothermality (bio2/bio7) (*100) (Isoth)
bio4	Temperature Seasonality (standard deviation*100)[excluded, *r* with bio7 = 0.9822]
bio5	Max Temperature of Warmest Month [excluded, *r* with bio10 = 0.9815]
bio6	Min Temperature of Coldest Month [excluded, *r* with bio11 = 0.9970]
bio7	Temperature Annual Range (TAR, bio5-bio6)
bio8	Mean Temperature of Wettest Quarter
bio9	Mean Temperature of Driest Quarter [excluded, *r* with bio11 = 0.9873]
bio10	Mean Temperature of Warmest Quarter (TWQ)
bio11	Mean Temperature of Coldest Quarter (TCQ)
bio12	Annual Precipitation
bio13	Precipitation of Wettest Month
bio14	Precipitation of Driest Month [excluded, *r* with bio19 = 0.9938]
bio15	Precipitation Seasonality (Coefficient of Variation)
bio16	Precipitation of Wettest Quarter [excluded, *r* with bio12 = 0.9547]
bio17	Precipitation of Driest Quarter [excluded, r with bio19 = 0.9910]
bio18	Precipitation of Warmest Quarter
bio19	Precipitation of Coldest Quarter

Supplementary Table 3 The out put of negative binomial generalized linear model for mountain species richness (SR).

	GLM		SEM	
Explantory variables	Coefficient	*t*	Coefficient	*z*
**landform model**				
Intercept	6.945	61.43***	6.899	59.86***
Desert	-0.456	-3.04***	-0.356	-2.22*
Granitic	0.290	2.42*	0.338	2.85**
Karst	0.155	1.11^{ns}	0.193	1.35^{ns}
Karst-Granitic	0.355	2.40*	0.415	2.75**
Deviance, %	28.95		31.40	
AIC	116.21		115	
Moran's I	0.096*		0.002^{ns}	
**full model**				
Intercept	6.707	34.20***	6.730	35.09***
Longitude	1.052	4.69***	1.033	4.36***
Elevdiff	1.121	4.70***	1.097	4.83***
TWQ	-1.137	-4.34***	-1.162	-4.51***
P_{var}	-0.616	4.40***	-0.614	-4.16***
Danxia:TCQ	0.693	3.00**	0.693	2.87**
Desert:TCQ	-2.788	-2.99**	-2.704	-3.01**
Granitic:TCQ	0.913	4.92***	0.938	4.72***
Karst:TCQ	0.959	4.56***	0.948	4.31***
Karst-Gr:TCQ	1.073	4.70***	1.122	4.66***
Deviance, %	62.8		63.71(62.69)	
AIC	35.75		35.39	
Moran's I	0.080^{ns}		0.004^{ns}	

The SR is log-transformed species richness. The landform effects were coded in reference to the Danxia landform (the intercept). We applied mixed-effects modelling approach (two-sided). The model was selected by stepwise procedure using the criterion of AIC. TWQ (Mean Temperature of Warmest Quarter); TCQ (Mean Temperature of Coldest Quarter); P_{var} (Precipitation Seasonality). ***p<.001; **p<.01; *p<.05; ns=not significant.

Supplementary Table 4 The out put of multiple regression models for PDI.

	GLM		SEM	
Explantory variables	Coefficient	*t*	Coefficient	*z*
**landform model**				
Intercept	-3.022	-3.53***	-3.536	-3.89***
Desert	-13.270	-11.65***	-9.256	-7.06***
Granitic	0.880	0.97^{ns}	1.139	1.36^{ns}
Karst	1.877	1.77^{ns}	1.711	1.62^{ns}
Karst-Granitic	1.113	0.99^{ns}	1.166	1.05^{ns}
Deviance, %	70.89		77.15 (70.85)	
AIC	683.21		667.89	
Moran's I	0.188***		-0.029^{ns}	
**full model**				
Intercept	15.405	2.93**	15.832	2.94**
Desert	-2.695	-2.47*	-2.062	-1.88^{ns}
Granitic	1.865	2.96**	2.007	3.29**
Karst	1.295	1.66ns	1.423	1.86^{ns}
Karst-Granitic	1.917	2.26*	2.001	2.41*
Orogenic	-1.417	-3.95***	-1.377	-3.66***
Latitude	-11.386	-5.04***	-11.294	-5.02***
TAR	-32.080	-4.51***	-33.889	-4.66***
TWQ	30.880	6.70***	32.407	6.80***
TCQ	-39.838	-4.73***	-41.484	-4.77***
Deviance, %	88.09		88.25 (88.04)	
AIC	568.51		569.40	
Moran's I	0.047^{ns}		0.002^{ns}	

The landform effects were coded in reference to the Danxia landform (the intercept). The effect of orogen (i.e., tectonic effect) is in reference to craton. We applied mixed-effects modelling approach (two-sided). The model was selected by stepwise procedure using the criterion of AIC. TAR (Temperature Annual Range); TWQ (Mean Temperature of Warmest Quarter); TCQ (Mean Temperature of Coldest Quarter). ***p<.001; **p<.01; *p<.05; ns=not significant.

Supplementary Table 5 The output of multiple regression models for MDT.

	GLM		SEM	
Explantory variables	Coefficient	*t*	Coefficient	*z*
**landform model**				
Intercept	7.848	39.48***	7.801	39.78***
Desert	-0.873	-3.30**	-0.112	-0.43^{ns}
Granitic	0.778	3.70***	0.821	5.33***
Karst	1.038	4.23***	0.931	4.70***
Karst-Granitic	0.962	3.70***	0.842	4.03***
Deviance, %	42.78		71.76 (landform only 39.38)	
AIC	274.24		205.79	
Moran's I	0.509***		-0.040^{ns}	
**full model**				
Intercept	12.137	9.76***	12.197	9.40***
Desert	0.629	2.41*	0.638	2.43*
Granitic	0.663	4.42***	0.682	4.70***
Karst	0.799	4.58***	0.814	4.74***
Karst-Granitic	0.775	4.00***	0.768	4.04***
Latitude	-2.098	-3.89***	-2.190	-4.03***
Elevdiff	0.980	2.67**	0.929	2.62**
TAR	-6.5101	-3.86***	-6.508	-3.71***
TWQ	3.7196	3.32**	3.890	-3.31***
TCQ	-6.1052	2.01**	-6.359	-3.00**
Deviance, %	75.52		76.38 (75.44)	
AIC	165.36		164.21	
Moran's I	0.089*		0.010^{ns}	

The landform effects were coded in reference to the Danxia landform (the intercept). We applied mixed-effects modelling approach (two-sided). The model was selected by stepwise procedure using the criterion of AIC. TAR (Temperature Annual Range); TWQ (Mean Temperature of Warmest Quarter); TCQ (Mean Temperature of Coldest Quarter). ***p<.001; **p<.01; *p<.05; ns=not significant.

Supplementary Table 6 The output of multiple regression models for MDT.oldest.

	GLM		SEM	
Explantory variables	Coefficient	*t*	Coefficient	*z*
**landform model**				
Intercept	22.049	44.21***	21.631	41.86***
Desert	-2.783	-4.20***	-0.678	-0.91ns
Granitic	2.120	4.02***	2.527	5.41**
Karst	2.370	3.85***	2.462	4.16***
Karst-Granitic	2.611	4.00***	2.551	4.08***
Deviance, %	49.08		62.83 (landform only 45.49)	
AIC	531.76		506.76	
Moran's I	0.272***		-0.012ns	
**full model**				
Intercept	25.588	32.57***	25.474	31.47***
Desert	0.909	1.22ns	1.242	1.61ns
Granitic	2.304	5.14***	2.460	5.61***
Karst	2.251	4.27***	2.337	4.43***
Karst-Granitic	2.808	5.08***	2.757	4.99***
Latitude	-3.579	-2.66**	-4.150	-2.97**
Isothermality	-2.561	-3.17**	-2.636	-3.01**
TAR	-4.347	-2.46*	-3.824	-2.24*
Deviance, %	65.69		67.05 (65.52)	
AIC	482.49		480.88	
Moran's I	0.096*		0.008ns	

The landform effects were coded in reference to the Danxia landform (the intercept). We applied mixed-effects modelling approach (two-sided). The model was selected by stepwise procedure using the criterion of AIC. TAR (Temperature Annual Range). ***p<.001; **p<.01; *p<.05; ns=not significant.

Supplementary Table 7 The output of multiple regression models for MDT$_{.youngest.}$

	GLM		SEM	
Explantory variables	Coefficient	*t*	Coefficient	*z*
**landform model**				
Intercept	0.663	13.72***	0.685	14.76***
Desert	-0.183	-2.86**	-0.061	-1.40^{ns}
Granitic	0.064	1.25^{ns}	0.034	1.38^{ns}
Karst	0.150	2.52*	0.107	3.38***
Karst-Granitic	0.078	1.23^{ns}	0.061	1.82^{ns}
Deviance, %	23.20		84.04(21.01)	
AIC	-122.08		-292.75	
Moran's I	0.775***		-0.088*	
**full model**				
Intercept	2.566	8.06***	1.500	4.50***
Desert	0.190	3.70***	0.054	1.15^{ns}
Granitic	0.034	1.15^{ns}	0.017	0.73^{ns}
Karst	0.094	2.52*	0.097	3.28**
Karst-Granitic	0.059	1.47^{ns}	0.068	2.09*
Orogenic	0.058	3.39***	0.015	0.72^{ns}
Latitude	-0.412	-3.73***	-0.154	-1.30^{ns}
Isothermality	0.561	6.24***	0.100	0.93^{ns}
TAR	-3.328	-7.08***	-1.445	-2.79**
TWQ	2.370	6.37***	0.773	1.90^{ns}
TCQ	-3.813	-6.03***	-1.262	-1.84^{ns}
Deviance, %	78.24		86.45(69.65)	
AIC	-286.65		-323.21	
Moran's I	0.249***		-0.049^{ns}	

The landform effects were coded in reference to the Danxia landform (the intercept). We applied mixed-effects modelling approach (two-sided). The model was selected by stepwise procedure using the criterion of AIC. TAR (Temperature Annual Range); TWQ (Mean Temperature of Warmest Quarter); TCQ (Mean Temperature of Coldest Quarter). ***p<.001; **p<.01; *p<.05; ns=not significant.

Supplementary Table 8 The out put of multiple regression models for NRI.

	GLM		SEM	
Explantory variables	Coefficient	*t*	Coefficient	*z*
**landform model**				
Intercept	-0.897	-1.21^{ns}	0.181	0.22^{ns}
Desert	10.779	-10.97***	4.856	4.41***
Granitic	-0.098	-0.13^{ns}	-0.820	-1.25^{ns}
Karst	0.392	0.43^{ns}	0.369	0.445^{ns}
Karst-Granitic	0.538	0.56^{ns}	-0.072	-0.08^{ns}
Deviance, %	64.91		77.43 (61.80)	
AIC	641.92		611.16	
Moran's I	0.221*		-0.056^{ns}	
**full model**				
Intercept	-15.180	-2.70**	-15.735	-2.60**
Desert	2.794	2.40*	1.406	1.22^{ns}
Granitic	-0.549	-0.86^{ns}	-0.791	-1.31^{ns}
Karst	0.032	0.04^{ns}	0.213	0.29^{ns}
Karst-Granitic	-0.345	-0.41^{ns}	-0.363	-0.45^{ns}
Longitude	-4.525	-2.98**	-5.140	-2.89**
Latitude	10.077	4.33***	10.232	4.30***
TAR	24.221	3.32**	26.682	3.43***
TWQ	-18.162	-3.66***	-19.272	-3.59***
TCQ	29.495	3.34**	31.057	3.23**
Deviance, %	80.01		81.51 (79.58)	
AIC	573.12		568.01	
Moran's I	0.136^{ns}		-0.005^{ns}	

The landform effects were coded in reference to the Danxia landform (the intercept). The effect of orogen (i.e., tectonic effect) is in reference to craton. We applied mixed-effects modelling approach (two-sided). The model was selected by stepwise procedure using the criterion of AIC. TAR (Temperature Annual Range); TWQ (Mean Temperature of Warmest Quarter); TCQ (Mean Temperature of Coldest Quarter). ***p<.001; **p<.01; *p<.05; ns=not significant.

Supplementary Table 9 The out put of multiple regression models for NTI.

	GLM		SEM	
Explantory variables	Coefficient	*t*	Coefficient	*z*
**landform model**				
Intercept	4.987	8.34***	4.886	7.91***
Desert	6.062	7.62***	5.019	5.64***
Granitic	-1.297	-2.05*	-1.135	-1.86^{ns}
Karst	-1.871	-2.53*	-1.545	-2.05*
Karst-Granitic	-1.486	-1.90^{ns}	-1.085	-1.36^{ns}
Deviance, %	57.88		62.71 (57.81)	
AIC	582.49		574.53	
Moran's I	0.163**		-0.013^{ns}	
**full model**				
Intercept	-11.404	-2.36*	-11.283	-2.32*
Desert	0.587	0.64	0.384	0.42^{ns}
Granitic	-1.795	-3.36**	-1.802	-3.50***
Karst	-0.855	-1.28	-0.936	-1.45^{ns}
Karst-Granitic	-1.326	-1.84	-1.337	-1.91^{ns}
Orogenic	0.869	2.86**	0.856	2.77**
Latitude	8.057	3.70***	7.958	3.70***
TAR	23.394	3.80***	23.706	3.84***
TWQ	-19.777	-4.82***	-20.146	-4.88***
TCQ	26.993	3.64***	27.344	3.65***
PREC	3.252	3.02**	3.041	2.86**
Deviance, %	75.03		75.19 (75.00)	
AIC	521.27		522.74	
Moran's I	0.034^{ns}		0.002^{ns}	

The landform effects were coded in reference to the Danxia landform (the intercept). The effect of orogen (i.e., tectonic effect) is in reference to craton. We applied mixed-effects modelling approach (two-sided). The model was selected by stepwise procedure using the criterion of AIC. TAR (Temperature Annual Range); TWQ (Mean Temperature of Warmest Quarter); TCQ (Mean Temperature of Coldest Quarter); PREC(Annual Precipitation). ***p<.001; **p<.01; *p<.05; ns=not significant.

Supplementary Table 10 Results of the interactions between landform and others variables on the species richness (SR).

Variables	GLM			SEM		
	Coefficient	SE	*t*	Coefficient	SE	*z*
TWQ	0.206	0.187	1.106ns	-1.072	0.229	-4.691***
TCQ	0.760	0.153	4.956***	0.668	0.179	3.733***
PREC	0.957	0.132	7.269***	0.968	0.151	6.428***
PCQ	0.498	0.117	4.275***	0.466	0.150	3.102***
Landform*TCQ (Intercept)	7.747	1.171	6.616***	8.141	1.130	7.2***
Desert	-0.513	1.183	-0.434ns	-0.859	1.142	-0.752ns
Granitic	-0.935	1.179	-0.793ns	-1.325	1.139	-1.163ns
Karst	-0.212	1.251	-0.169ns	-0.634	1.206	-0.526ns
Karst-Gr	1.020	1.324	0.771ns	0.399	1.283	0.311ns
TCQ	-1.113	1.620	-0.687ns	-1.724	1.573	-1.096ns
Desert:TCQ	-5.291	2.025	-2.613ns	-4.812	1.920	-2.506*
Granitic:TCQ	1.767	1.633	1.082*	2.367	1.587	1.492ns
Karst:TCQ	0.488	1.736	0.281ns	1.152	1.683	0.685ns
Karst-Gr:TCQ	-1.384	1.925	-0.719ns	-0.329	1.879	-0.175ns
Landform*PREC (Intercept)	7.765	0.997	6.784***	6.320	0.907	6.948***
Desert	-0.702	1.018	-0.689ns	-0.584	0.942	-0.620ns
Granitic	0.0153	1.006	0.015ns	0.523	0.918	0.570ns
Karst	-0.607	1.195	-0.509ns	-0.449	1.084	-0.414ns
Karst-Gr	0.517	1.088	0.475ns	1.008	0.980	1.029ns
PREC	0.274	1.505	0.182ns	0.888	1.371	0.648ns
Desert:PREC	3.950	2.368	1.668ns	6.115	2.372	2.578**
Granitic:PREC	0.408	1.516	0.269ns	-0.268	1.380	-0.194ns
Karst:PREC	1.497	1.941	0.771ns	1.381	1.766	0.782ns
Karst-Gr:PREC	-0.240	1.706	-0.140ns	-0.942	1.538	-0.612ns
Landform*PCQ (Intercept)	6.821	0.485	14.071***	6.643	0.474	14.005***
Desert	-0.765	0.511	-1.495ns	-0.563	0.501	-1.124ns
Granitic	0.231	0.491	0.471ns	0.391	0.480	0.816ns
Karst	0.148	0.572	0.259ns	0.292	0.554	0.527ns
Karst-Gr	0.690	0.516	1.338ns	0.899	0.499	1.801ns
PCQ	0.192	0.730	0.263ns	0.430	0.711	0.605ns
Desert:PCQ	13.196	4.178	3.158**	14.191	4.023	3.528***
Granitic:PCQ	0.159	0.742	0.214ns	-0.040	0.718	-0.055ns
Karst:PCQ	0.261	1.248	0.209ns	0.057	1.195	0.048ns
Karst-Gr:PCQ	-0.968	0.918	-1.055ns	-1.298	0.879	-1.480ns

The SR is log-transformed species richness. We applied mixed-effects modelling approach (two-sided). TWQ (Mean Temperature of Warmest Quarter); TCQ (Mean Temperature of Coldest Quarter); PCQ (Precipitation of Coldest Quarter). There are interaction effects between landform effect and other variables. ***p<.001; **p<.01; *p<.05; ns=not significant.

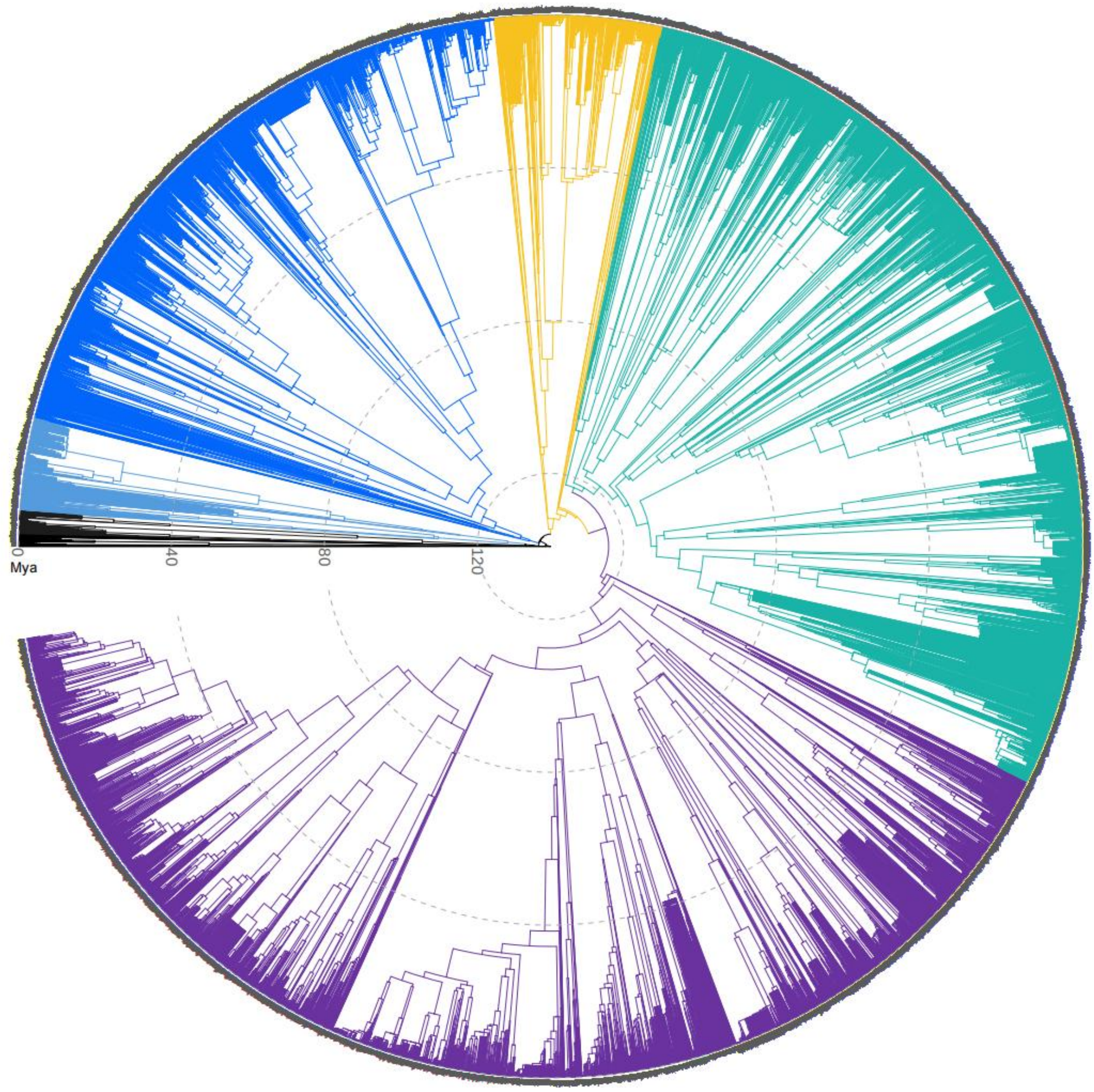

Supplementary Fig. 1 Dated megaphylogeny in this study. The color of branch represent major angiosperm clade based on APG IV (black, ANATA; light blue, magnoliids; , blue monocots; orange, basal eudicots; green, superrosids; purple, superasterids). Divergence times were estimated using GBOTB[138,139]. The original image file could be found at https://itol.embl.de/export/1836994402281644388267.

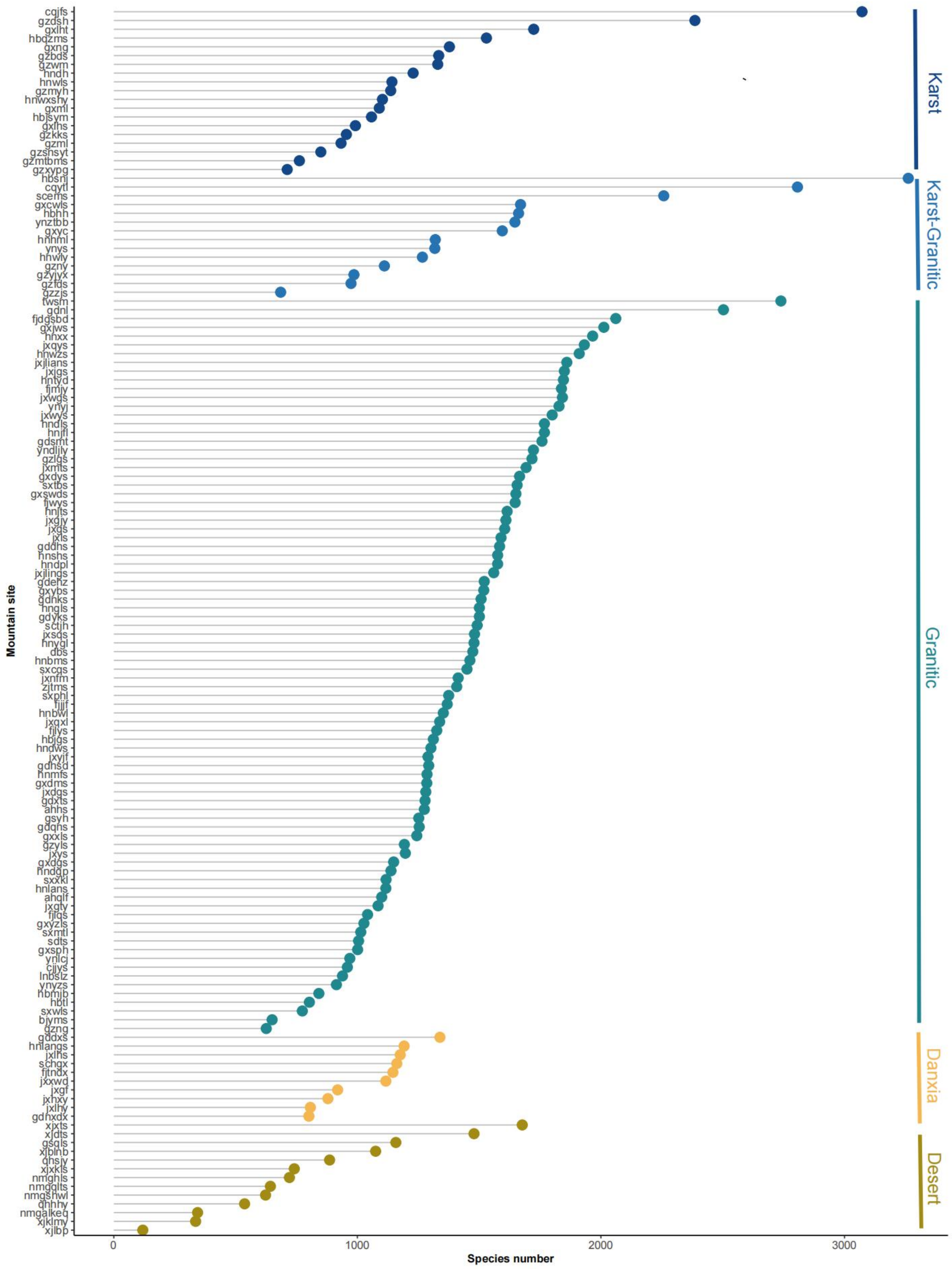

Supplementary Fig. 2 Species richness of the 140 mountain floras in this study. The sample sizes (*n*) for Danxia, Desert, Granitic, Karst, Karst-Granitic are 10, 13, 84, 19 and 14, respectively. Source data are provided as a Source Data file.

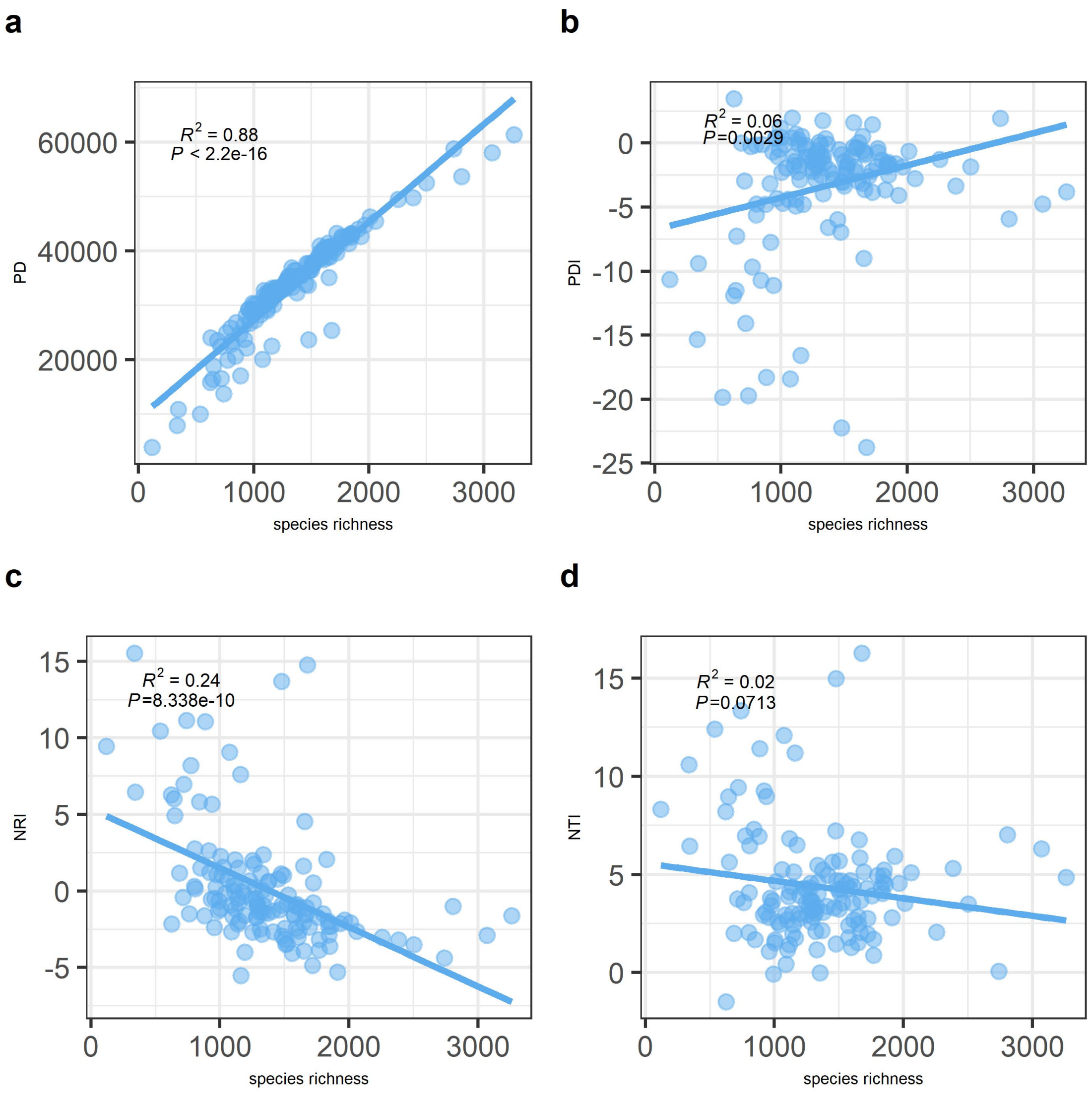

Supplementary Fig. 3 Regression analyses between species richness and PD, PDI, NRI, NTI. It shows a strong collinearity between species richness and PD (**a**, n=140), and weak collinearity to phylogenetic structure PDI (**b**, n=140), NRI (**c**, n=140), and NTI (**d**, n=140). PD, phylogenetic diversity; PDI, phylogenetic diversity index; NRI, net relatedness index; NTI, nearest taxon index. Relationships are denoted with solid lines and fit statistics (R2 and P values). Source data are provided as a Source Data file.

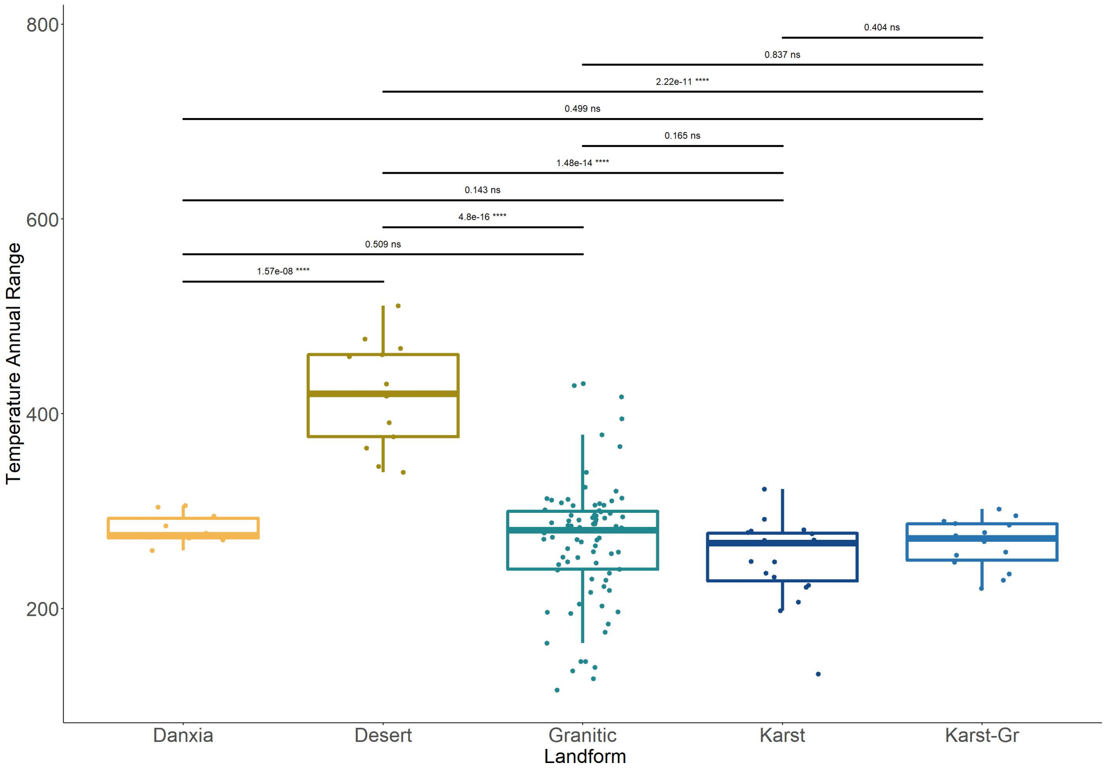

Supplementary Fig. 4 Temperature annual range (TAR) of different landforms. The sample sizes (*n*) for Danxia, Desert, Granitic, Karst, Karst-Gr are 10, 13, 84, 19 and 14, respectively. The box plots show the first and third quartiles (box limits), median (center line), and whiskers extend to a maximum of 1.5 times the interquartile range. Differences between each pair of landforms determined by using a two-sided, independent samples t test and *P*-values shown above the black line. ****P<.00001; ns = not significant. Source data are provided as a Source Data file.

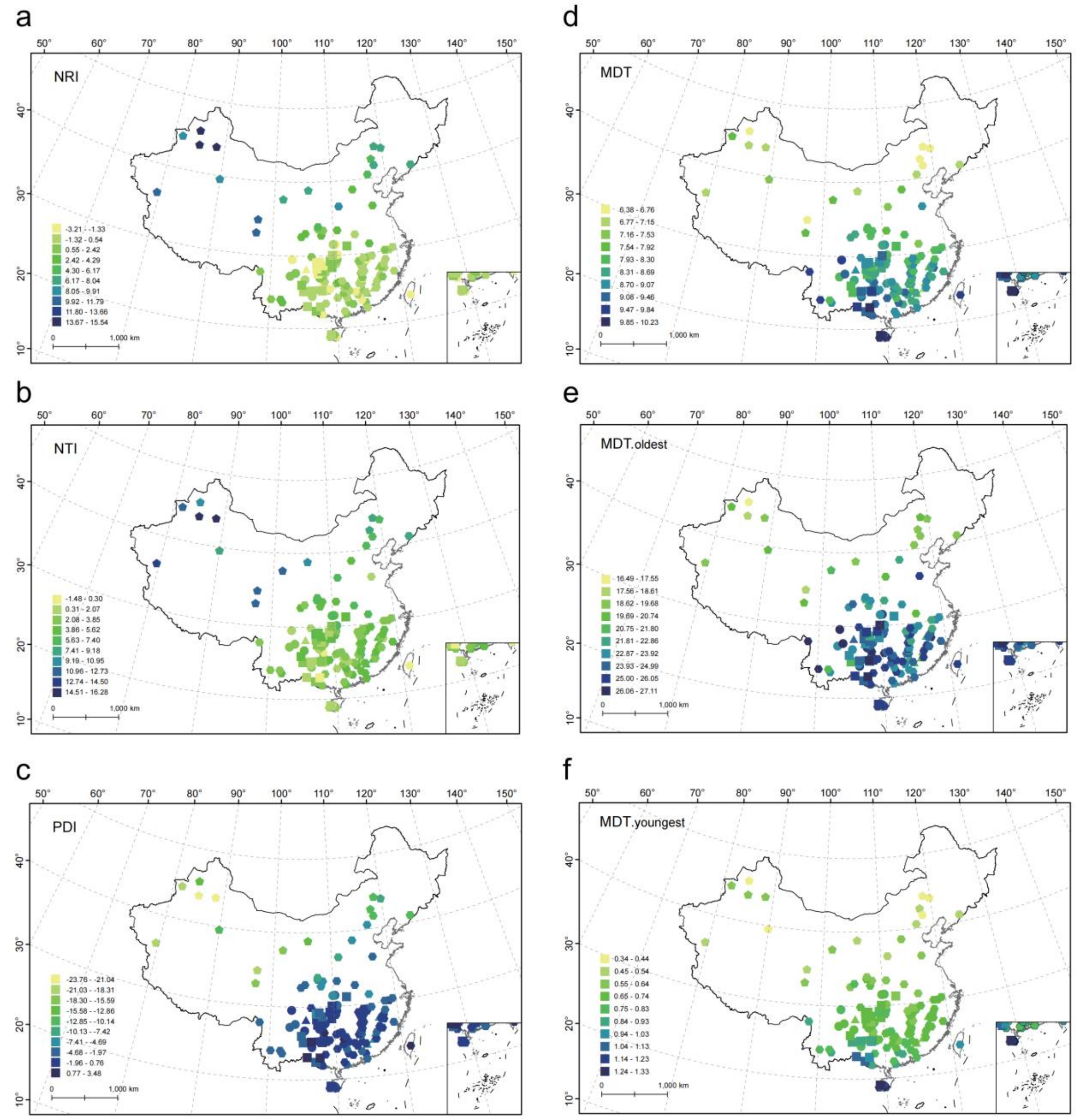

Supplementary Fig. 5 Geographic patterns of phylogenetic structure, and MDTs of 140 mountain floras. a NRI (net relatedness index), **b** NTI (nearest taxon index), **c** PDI (phylogenetic diversity index), **d** MDT (mean diversity time of all species), **e** MDT.$_{\text{oldest}}$ (mean divergence time of the oldest 25% of species), **f** MDT.$_{\text{youngest}}$ (mean divergence time of the youngest 25% of species). The shapes of mountain flora represents the type of landform, specifically the triangle is Danxia, square is karst, circle is karst-granitic, pentagon is desert, and hexagon is granitic. The sample sizes (*n*) for Danxia, desert, granitic, karst, karst-granitic are 10, 13, 84, 19 and 14, respectively. Source data are provided as a Source Data file.

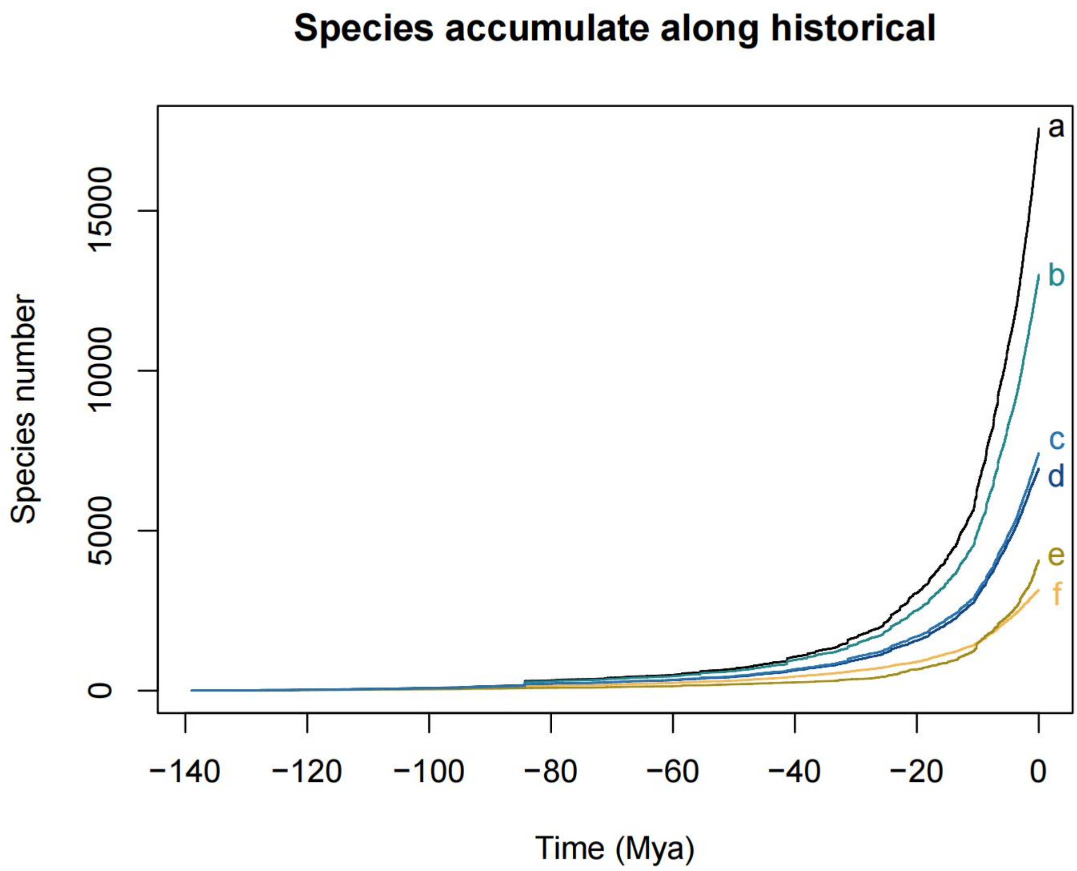

Supplementary Fig. 6 Number of angiosperm species of different landforms during specified geological times. **a** all species (*n*=17576); **b** species occurs in granitic landform (*n*=13005); **c** species occurs in karst-granitic landform (*n*=7423); **d** species occurs in karst landform (*n*=6941); **e** species occurs in desert landform (*n*=4077); **f** .species occurs in Danxia landform (*n*=3146). Source data of dated phylogenetic trees are available at https://doi.org/10.5061/dryad.b2rbnzsk1.

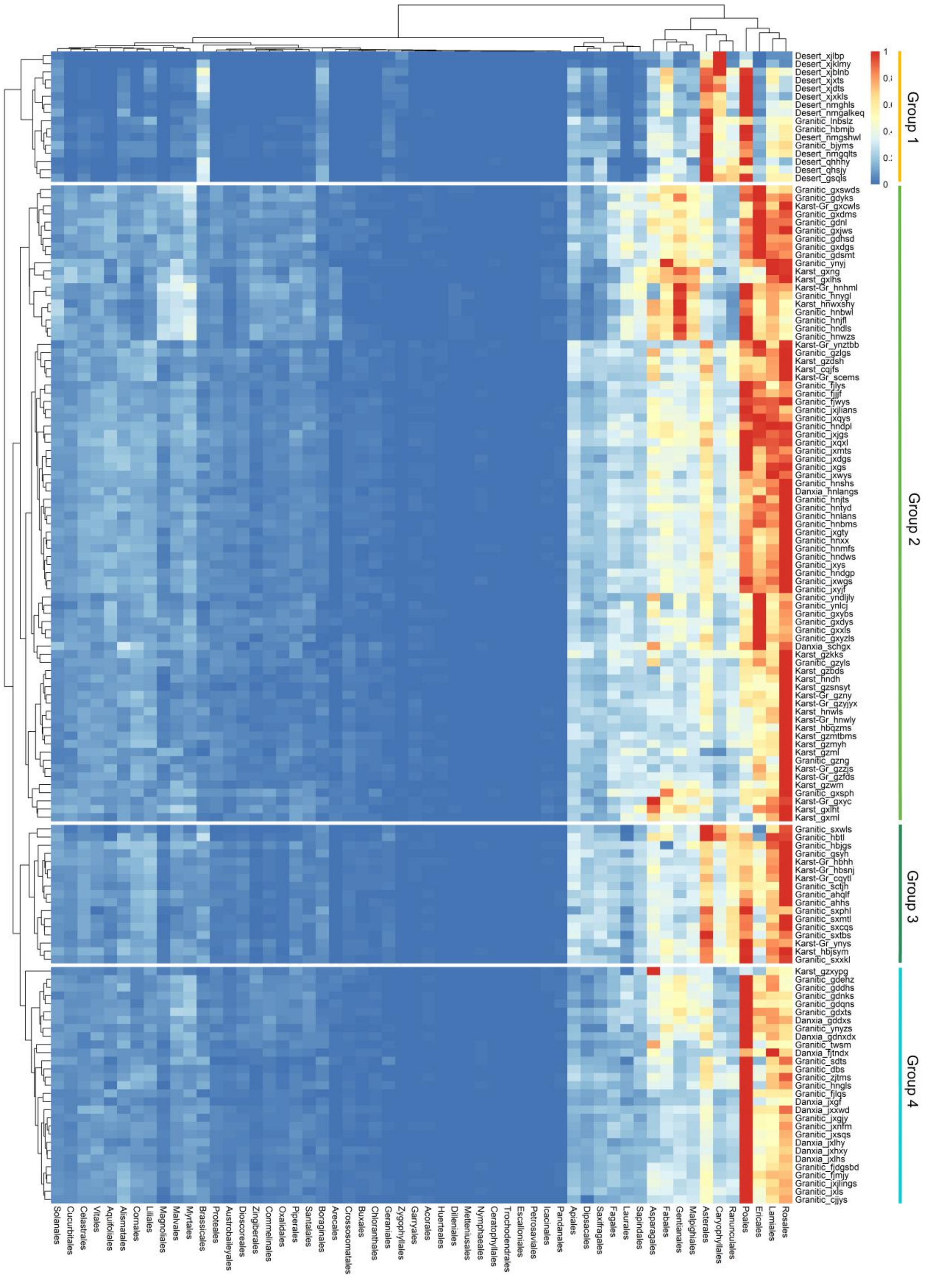

Supplementary Fig. 7 Thermodynamic chart of composition in order level of mountain floras. The species number of a order has been normalized to 0-1 range by $(x)/(x_{\max})$. It is shown that the flora composition of desert landform primarily belongs to the group 1, with a dominant presence of Poales, Asterales, Caryophyllales, Fabales, Brassicales, and Boraginales. The group 2 comprises Granitic, karst, and karst-granitic floras, which are rich in Rosales, Ericales, Poales, Lamiales, Asterales. Moreover, a subclade comprising karst and karst-granitic is nested within group two, with Ericales and Lamiales being less dominant than other subclades in this group. The group 3, located in central China, consists of granitic and karst-granitic floras. Among them, the Rosales exhibits the highest level of diversity, while Lamiales, Ericales, Asterales, and Asparagales are also abundant. The group 4 formed by mainly by Granitic and Danxia floras, there flora is abundant in Poales, Rosales, Lamiales, Ericales. Furthermore, Ericales in Danxia is less dominant than granitic, but Poales in Danxia is more abundant than granitic. Source data are provided as a Source Data file.

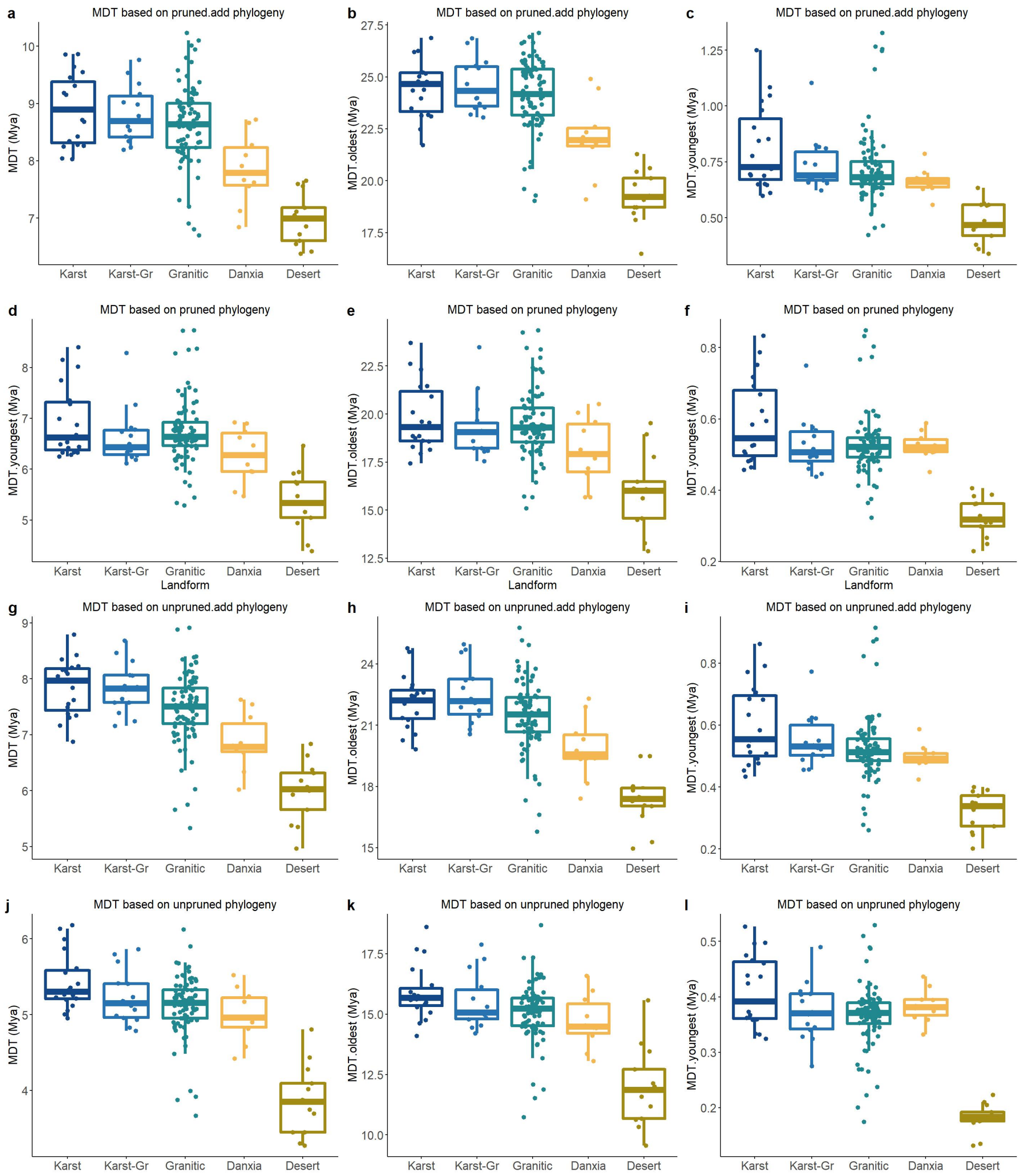

Supplementary Fig. 8 MDT results derived from four species divergence time datasets. a-c The species divergence time referred to a purned.add phylogenetic tree, encompassed a total of 17,576 species. Among these, ages for 8,863 species were extracted from the pruned phylogenetic tree, while ages for an additional 8,713 species (designated as add.age) were estimated using the BLADJ method[140]. **d-f** The unpurned.add phylogeny included 17,576 species, of which ages of 8863 species extract from the GBOTB.extended.LCVP.tre[139], and ages of others 8713 species (add.age) are added by BLADJ method[140]. **g-i** The purned phylogeny included 8863 species, age extract from the pruned phylogeny tree (this study). **j-l** The unpurned phylogeny included 8863 species, age extract from the GBOTB.extended.LCVP.tre[139]. Definition of MDT, MDT.youngest, and MDT.oldest are as in Supplementary Fig. 5. The results shown the MDT patterns of mountain flora are similar of the four different species divergence time datasets. The MDT of karst landform is always the highest, while the MDT of Danxia landform and desert landform is lower. The results also shown that the MDT estimate from pruned phylogeny (**j-l**) is higher than the unpruned phylogeny (**g-i**) , and the pruned.add phylogeny (**a-c**) is higher than the unpruned.add phylogeny (**d-f**). This could be expected, as the species branch in a pruned phylogeny is usually longer than which in the global plant phylogeny or a phylogeny included more species. In generally, the species ages inferred in this four phylogeny did not affect the MDT patterns between mountain in different landform. The sample sizes (*n*) for Danxia, desert, granitic, karst, karst-granitic are 10, 13, 84, 19 and 14, respectively. Differences between each pair of landforms determined by using a two-sided, independent samples *t* test. Source data are provided as a Source Data file.

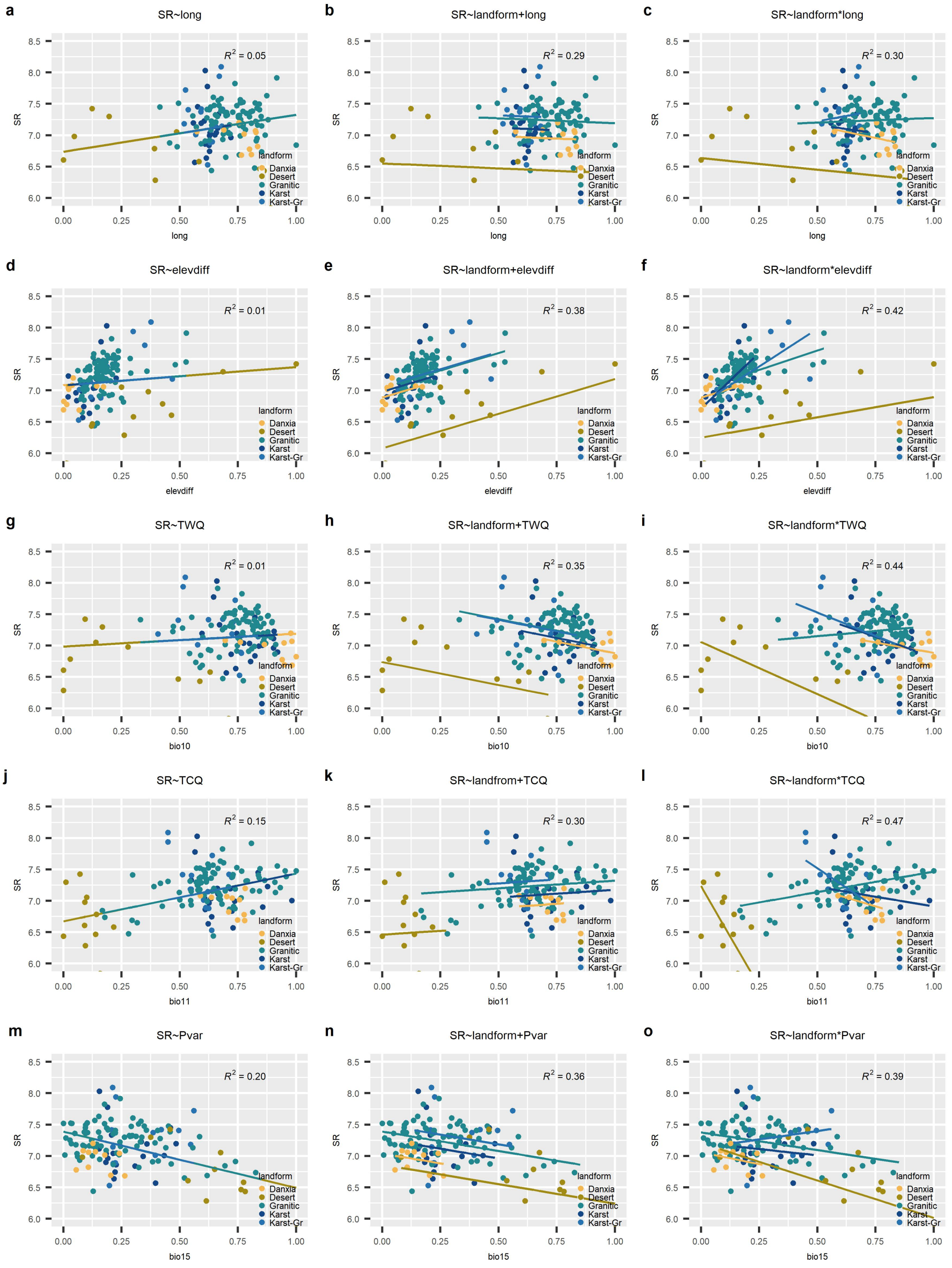

Supplementary Fig. 9 Interaction effects between landform and environmental variables on species richness (SR). a-c Interaction effects between landform and longitude on SR. **d-f** Interaction effects between landform and elevdiff (difference value between the highest elevation and lowest elevation) on SR. **g-i** Interaction effects between landform and TWQ (Mean Temperature of Warmest Quarter) on SR. **j-l** TCQ is positively correlated with SR (**j**). When consider the interactions with landform, the TCQ still positive correlation to the SR in granitic landform mountain, while negative correlation in Danxia, desert, and karst landfroms (**l**). **m-o** Interaction effects between landform and Pvar (Precipitation Seasonality) on SR. Here only shown the interaction of landfrom with the environmental variables, which occurs in the full model. Karst-Gr, karst-granitic. SR represent the log-converted species richness, and the other variables are normalization into 0-1 by formula $(x - x_{\min})/(x_{\max} - x_{\min})$. Source data are provided as a Source Data file.

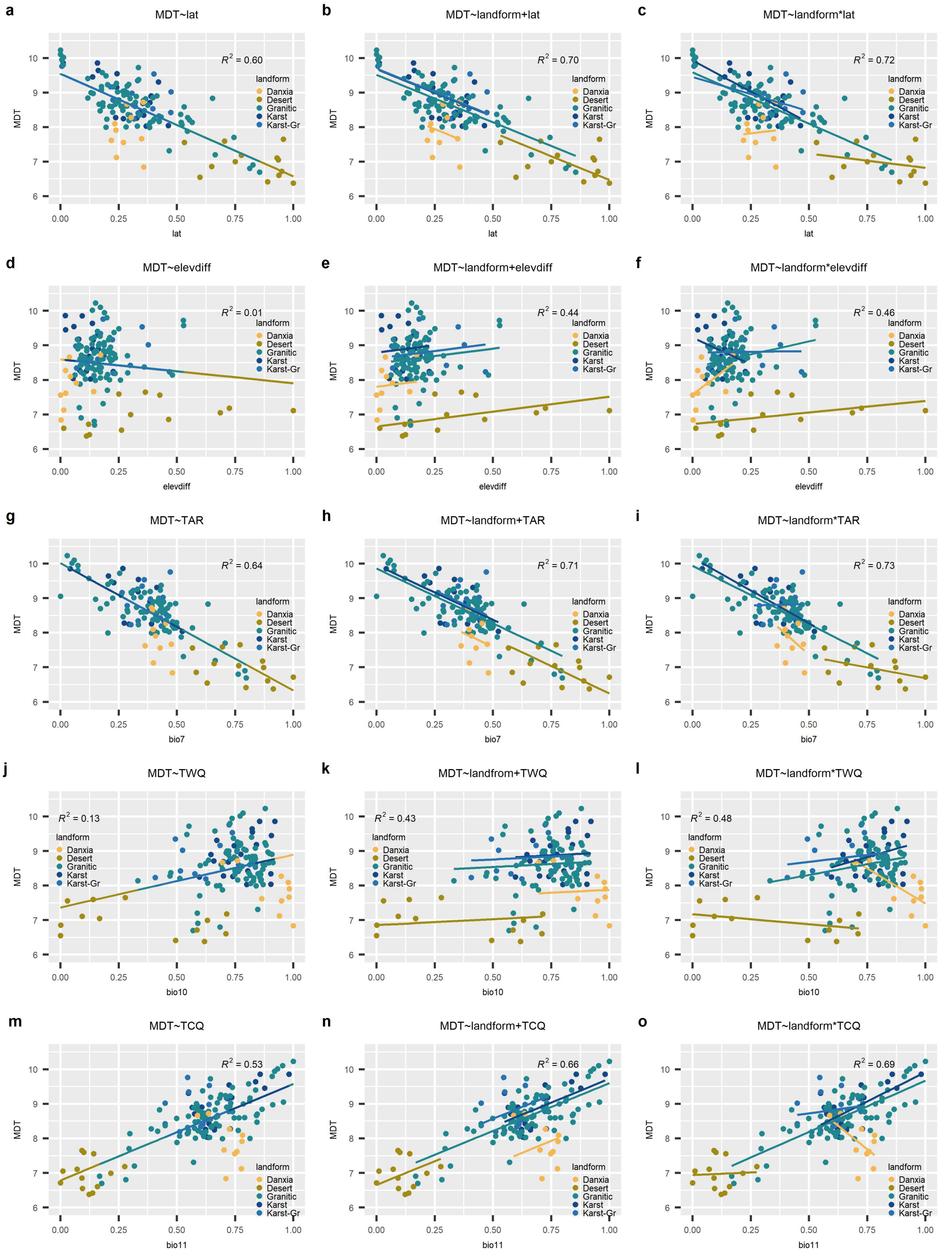

Supplementary Fig. 10 | Interaction effects between landform and environmental variables on MDT (mean diversity time of all species in a mountain). a-c Interaction effects between landform and lat (latitude) on MDT. **d-f** Interaction effects between landform and elevdiff (difference value between the highest elevation and lowest elevation) on MDT. **g-i** Interaction effects between landform and TAR (Temperature Annual Range) on MDT. **j-l** Interaction effects between landform and TWQ (Mean Temperature of Warmest Quarter) on MDT. **m-o** Interaction effects between landform and TCQ (Mean Temperature of Coldest Quarter) on MDT. Here only shown the interaction of landfrom with the environmental variables, which occurs in the full model. Karst-Gr, karst-granitic. Source data are provided as a Source Data file.

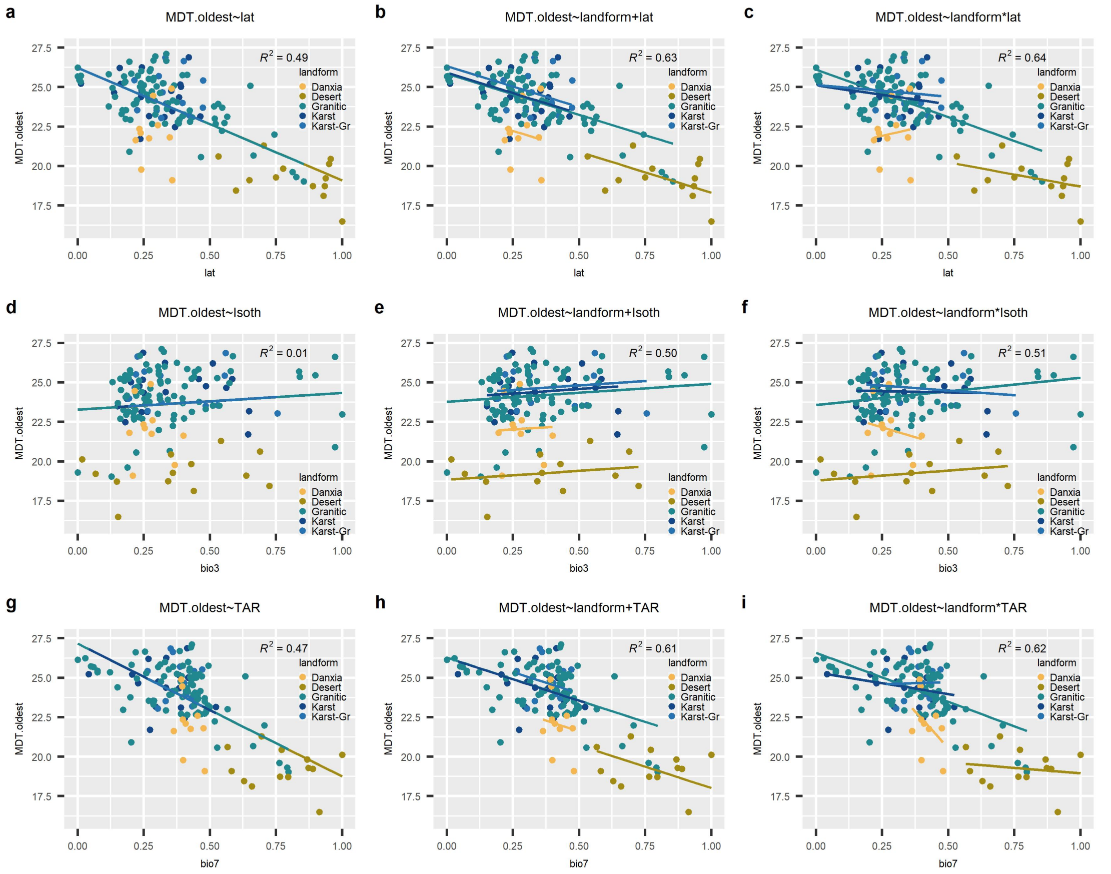

Supplementary Fig. 11 | Interaction effects between landform and environmental variables on MDT.oldest (mean diversity time of 25% oldest species in a mountain). a-c Interaction effects between landform and lat (latitude) on MDT.oldest. **d-f** Interaction effects between landform and Isoth (Isothermality) on MDT.oldest. **g-i** Interaction effects between landform and TAR (Temperature Annual Range) on MDT.oldest. Here only shown the interaction of landfrom with the environmental variables, which occurs in the full model. Karst-Gr, karst-granitic. Source data are provided as a Source Data file.

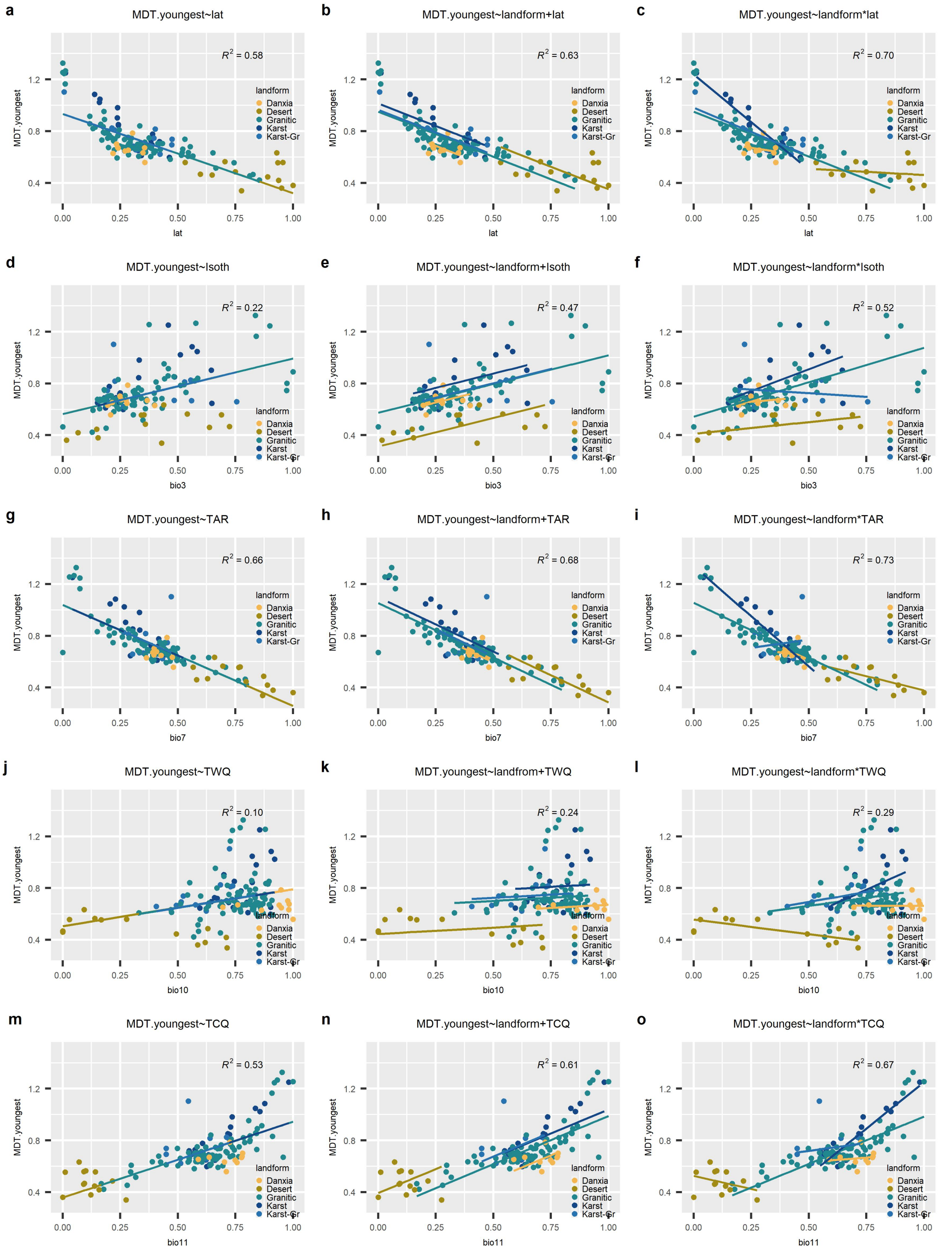

Supplementary Fig. 12 | Interaction effects between landform and environmental variables on MDT.youngest (mean diversity time of 25% youngest species in a mountain). a-c Interaction effects between landform and lat (latitude) on MDT.youngest. **d-f** Interaction effects between landform and Isoth (Isothermality) on MDT.youngest. **g-i** Interaction effects between landform and TAR (Temperature Annual Range) on MDT.youngest. **j-l** Interaction effects between landform and TWQ (Mean Temperature of Warmest Quarter) on MDT.youngest. **m-o** Interaction effects between landform and TCQ (Mean Temperature of Coldest Quarter) on MDT.youngest. Here only shown the interaction of landfrom with the environmental variables, which occurs in the full model. Karst-Gr, karst-granitic. Source data are provided as a Source Data file.

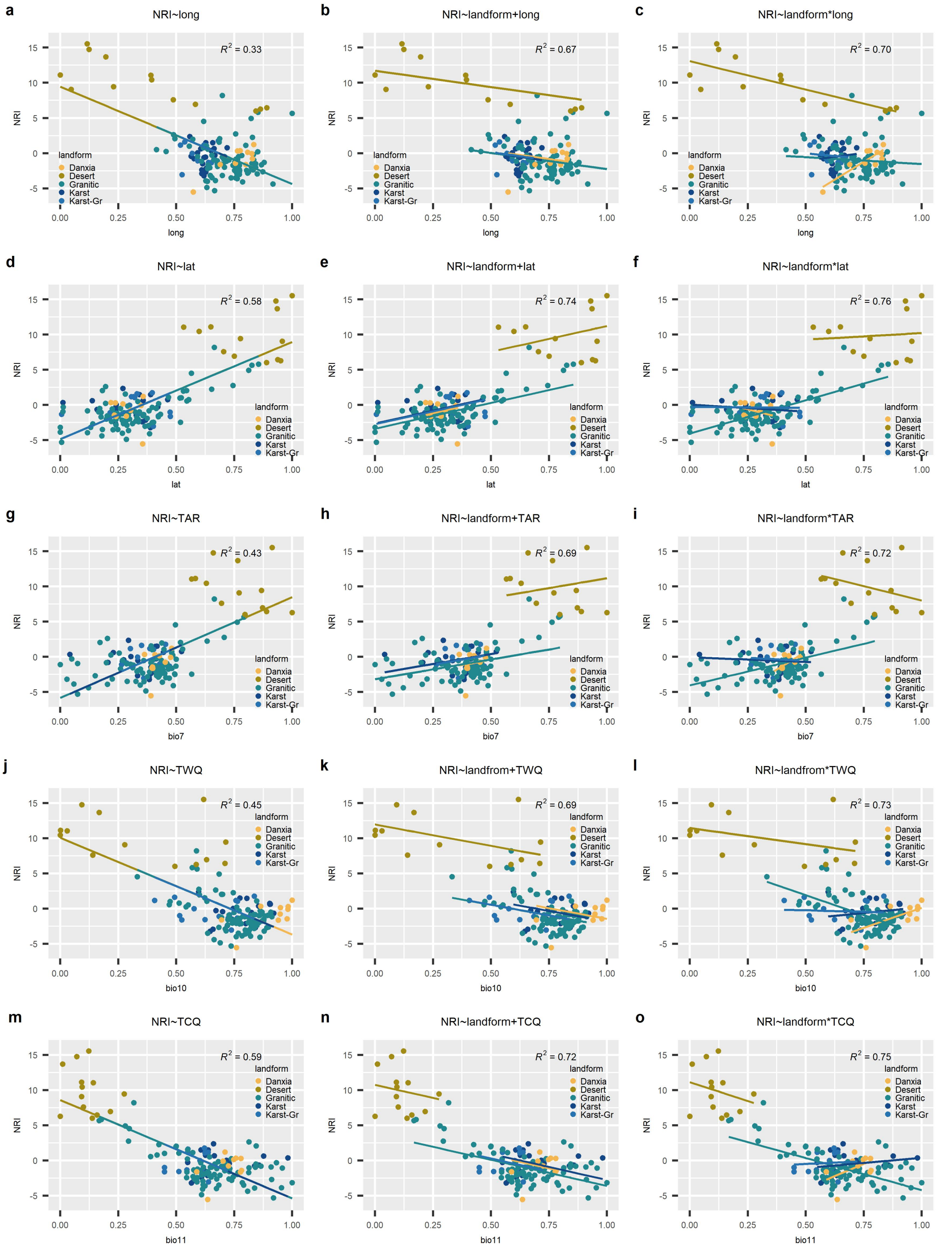

Supplementary Fig. 13 | Interaction effects between landform and environmental variables on NRI (net relatedness index). a-c Interaction effects between landform and long (longitude) on NRI. **d-f** Interaction effects between landform and lat (latitude) on NRI. **g-i** Interaction effects between landform and TAR (Temperature Annual Range) on NRI. **j-l** Interaction effects between landform and TWQ (Mean Temperature of Warmest Quarter) on NRI. **m-o** Interaction effects between landform and TCQ (Mean Temperature of Coldest Quarter) on NRI. Here only shown the interaction of landfrom with the environmental variables, which occurs in the full model. Karst-Gr, karst-granitic. Source data are provided as a Source Data file.

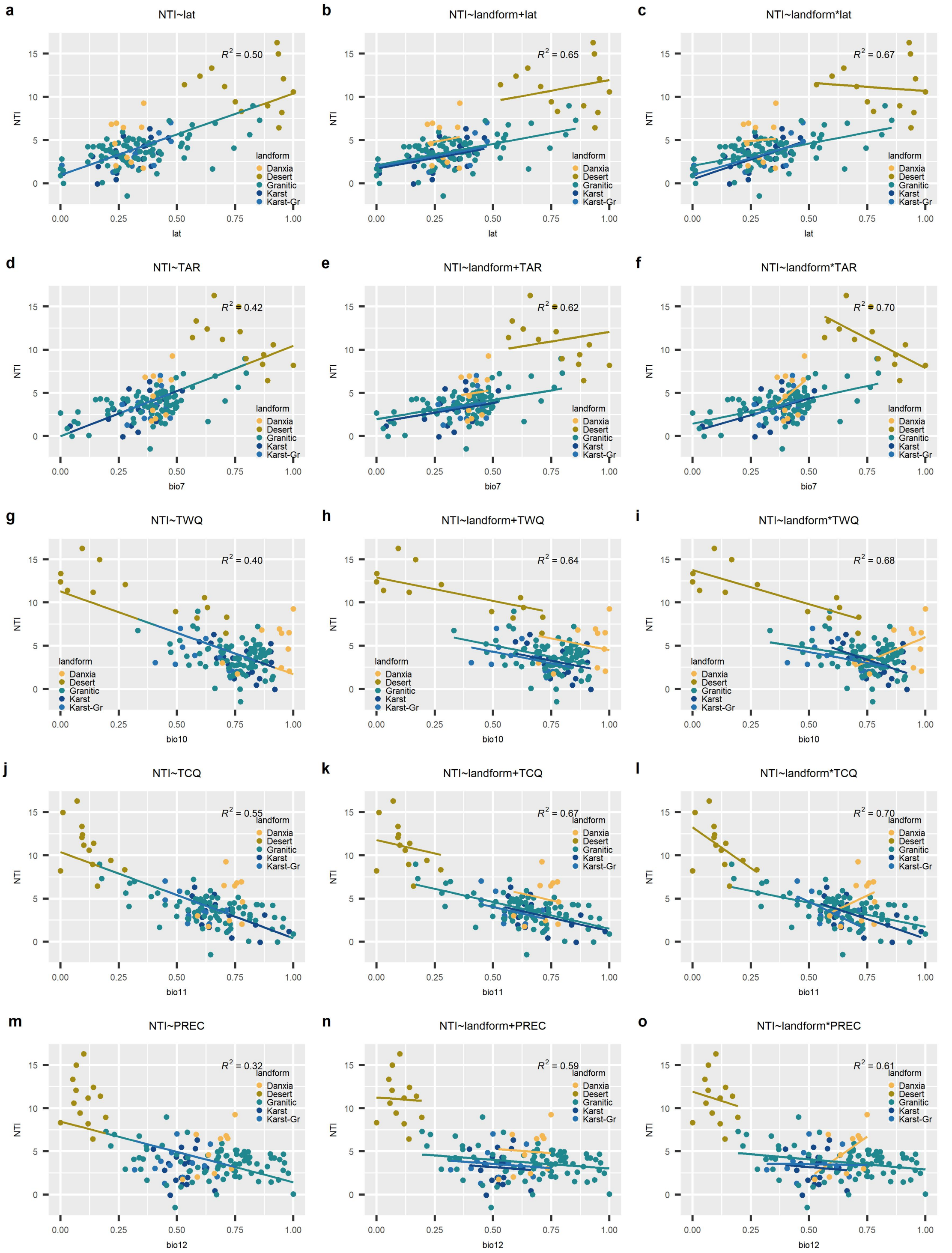

Supplementary Fig. 14 | Interaction effects between landform and environmental variables on NTI (nearest taxon index). a-c Interaction effects between landform and lat (latitude) on NTI. **d-f** Interaction effects between landform and TAR (Temperature Annual Range) on NTI. **g-i** Interaction effects between landform and TWQ (Mean Temperature of Warmest Quarter) on NTI. **j-l** Interaction effects between landform and TCQ (Mean Temperature of Coldest Quarter) on NTI. **m-o** Interaction effects between landform and PREC (Annual Precipitation) on NTI. Here only shown the interaction of landfrom with the environmental variables, which occurs in the full model. Karst-Gr, karst-granitic. Source data are provided as a Source Data file.

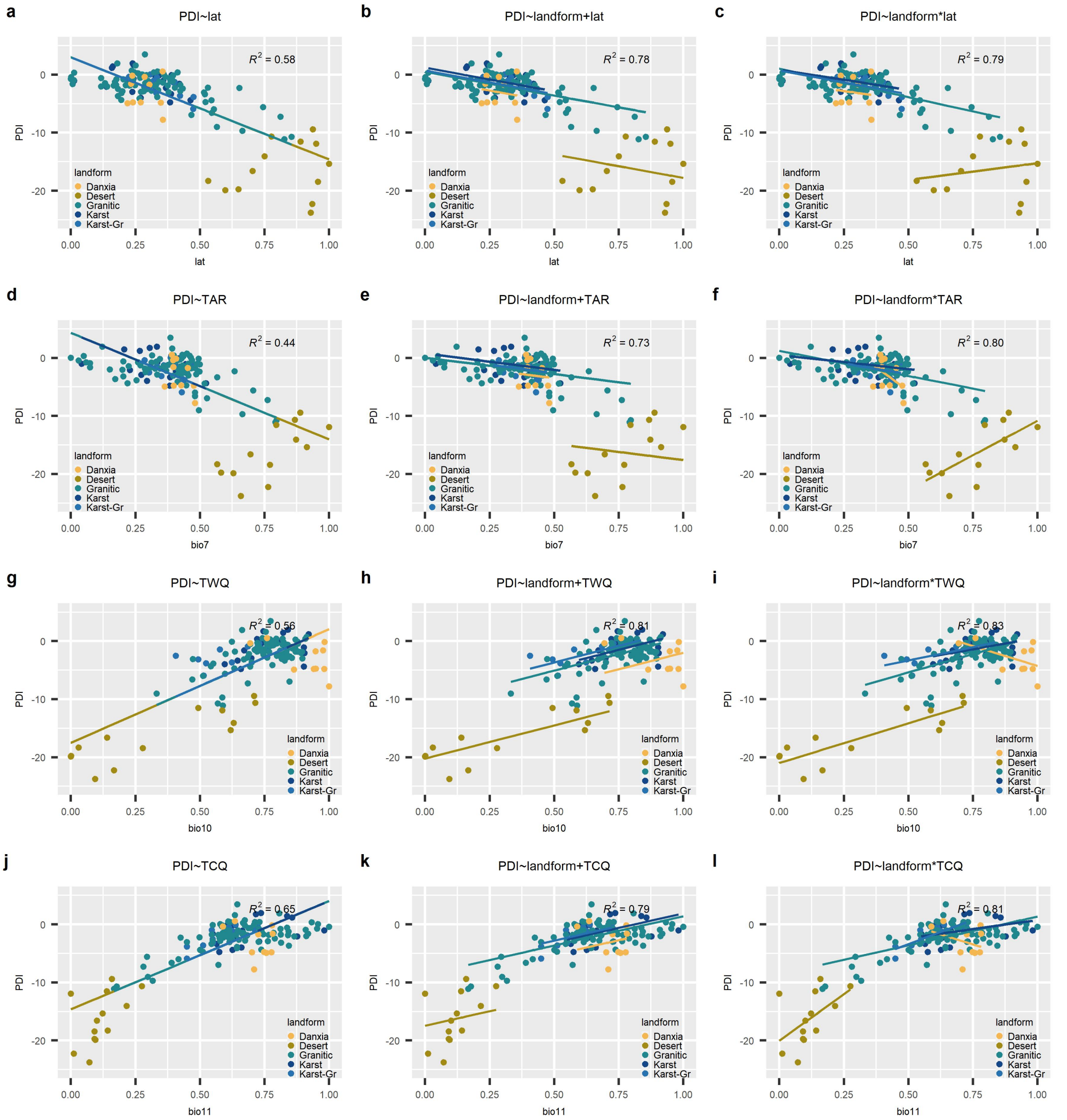

Supplementary Fig. 15 | Interaction effects between landform and environmental variables on PDI (phylogenetic diversity index). a-c Interaction effects between landform and lat (latitude) on PDI. **d-f** Interaction effects between landform and TAR (Temperature Annual Range) on PDI. **g-i** Interaction effects between landform and TWQ (Mean Temperature of Warmest Quarter) on PDI. **j-l** Interaction effects between landform and TCQ (Mean Temperature of Coldest Quarter) on PDI. Here only shown the interaction of landfrom with the environmental variables, which occurs in the full model. Karst-Gr, karst-granitic. Source data are provided as a Source Data file.

Supplementary Reference

1. Xu, K.-K. *Study on flora of Danxia Landform in Jiangxi* (Gannan Normal University, master dissertation, 2016).
2. Peng, S.-L., et al. *Comprehensive Scientific Investigation of Animal and Plant Resources in Danxiashan in Guangdong* (Science Press, 2011).
3. Hu, C. *A study of plant diversity evaluation in Danxia Wutong Nature Reserve* (Central South University of Forestry and Technology, master dissertation, 2018).
4. China Danxia World Natural Heritage Professional Protection Committee. *Plant Checklist of Langshan*. (Scientific Report, 2008).
5. Li, Z.-J., Chen, X.-L. & Liu, C.-M. *The Biodiversity of Taining World Natural Heritage* (Science Press, 2012).
6. Yang, Y.-C. *Report of Comprehensive Scientific Investigation of Huagaoxi Nature Reserve* (Sichuan Natural Resources Institute, 2001).
7. He, Y.-J. *Studies on Seed Plant Biodiversity and Conservation in Main Forest Regions of San Jiangyuan Nature Reserve, Qinghai Province* (Beijing Forestry University, Phd dissertation, 2004).
8. Wu, Y.-H. & Mei, L.-J. *Plant Resources and Environment of the Yellow River Source Area* (Qinghai Peoples Publishing House, 2001).
9. Zhang, L.-J. *Study on the Flora and Species Diversity of Plants in the West Kunlun Mountain in Xinjiang* (Shihezi University, master dissertation, 2018).
10. Liu, J.-Q., Yang, Q.-S. & Wang, Y.-K. C*omprehensive Investigation Report of Qilianshan National Nature Reserve in Gansu Province* (Gansu Science & Technology Press, 2008).
11. Liu, Z.-S. *Comprehensive Scientific Investigation Report of the Helanshan National Nature Reserve in Inner Mongolia* (Ningxia People's Publishing House, 2015).
12. Yuan, G.-Y., Zhang, Y. & Yuan, L. *Lopnur Nature Reserve* (Science Press, 2012).
13. Zhao, Y.-Y. *Study on the Flora of Seed Plants in the Qilaotu Mountain of Northern Yanshan Mountain* (Inner Mongolia Agricultural University, master dissertation, 2016).
14. Xiong, J.-W. *Comprehensive Scientific Investigation of the Western Tianshan Mountains in Xinjiang* (China Forestry Publishing House, 2017).
15. Xiong, J.-W. *Comprehensive Scientific Investigation of the Eastern Tianshan Mountain in Xinjiang* (China Forestry Publishing House, 2015).
16. Soyolt. *Study on Plants Diversity and Ethnobotany in Arhorchin National Nature Reserve* (Inner Mongolia Agricultural University, Phd dissertation, 2013).
17. Chen, T., Cui, D.-F. & Liao, W.-B. *Scientific Investigation of Mountain Forest Resources of Southern Bortala Mengol Autonomous Prefecture in Xinjiang* (China Forestry Publishing House, 2021).
18. Zhang, S.-L. *Study on Plants Diversity and Conservation in Saihanwula Nature Reserve Inner Mongolia* (Beijing Forestry University, Phd dissertation, 2007).
19. Lu, G.-H., et al. *Xinjiang Kelamayi Shengwu Duoyangxing Ji Baohu* (Xinjiang People's Publishing House, 2008).
20. Xu, Z. *Flora and Phytogeography of Mt. Houmiling Nature Reserve, Hainan, China* (South China Agricultural University, master dissertation, 2017).
21. Tan, W.-F. *Study on Biodiversity Conservation in Guangxi Cenwanglaoshan Nature Reserve* (China Environment Science Press, 2005).
22. Li, S.-W. *Study on Species Diversity of Vascular Plants in Yachang Orchid National Nature Reserve of Guangxi* (Guangxi Normal University, master dissertation, 2017).
23. Deng, L.-X., et al. *Scientific Survey and Research on Nayong Nature for Dove Tree in Guizhou province* (China Forestry Publishing House, 2013).

24. Xie, S.-X., Li, M.-J. & Yu, L.-F. *Scientific Survey of the Zhujiangshan Nature Reserve in Guizhou* (China Forestry Publishing House, 2000).
25. Peng, M.-C., Wang, C.-Y. & Dang, C.-L. *Biodiversity and Conservation in Yaoshan Nature Reserve, Yunnan* (Science Press, 2006).
26. Yu, L.-F., Li, M.-J. & Xie, S.-X. *Scientific Survey of the Fodingshan Nature Reserve in Guizhou* (China Forestry Publishing House, 2000).
27. Ding, L. *A Study on Floristic Phytogeography of Seed Plants in North of Chaotong Nature Reserve, Yunnan, China* (Southwest Forestry College, master dissertation, 2007).
28. Zhu, J., et al. *Comprehensive Scientific Survey and Research on Yangxi Nature Reserve in Yinjiang County of Guizhou Province* (China Forestry Publishing House, 2017).
29. Zhou, H. *The Research of Plant Diversity in Wulingyuan World Natural Heritage Site* (Central South University of Forestry and Technology, master dissertation, 2016).
30. Li, Z.-Y. & Shi, L. *Plants of Mount Emei* (Beijing Science and Technology Press, 2007).
31. Song, C.-S. & Liu, S.-X. *Scientific Survey of the Hubei Houhe Nature Reserve* (China Forestry Publishing House, 1999).
32. Gao, X.-M., Xu, W.-T. & Xie, Z.-Q. *Plant Checklist of Shennongjia* (Science Press, 2019).
33. Bi, R.-C., Wei, X.-Z., Duan, Z.-S., Wei, W.-L. & Cui, B.-Y. *Scientific Investigation Report of the Wulushan Nature Reserve in Shanxi* (China Science and Technology Press, 2004).
34. Huang, K.-Y. *Studies on the Spermatophytic Diversity of Diaoluoshan Nature Reserve in Hainan Island* (SunYat-Sen University, master dissertation, 2004).
35. Hainan Forestry Department, Hainan Jianfengling Nature Reserve Management Station, Nature Reserve Research Center of Beijing Forestry University. *Comprehensive Investigation Report of Jianfengling Nature Reserve in Hainan*. (Scientific Report, 2001).
36. Fan, Q. *Studies on the Flora of Wuzhishan Area in Hainan Island* (SunYat-sen University, master dissertation, 2004).
37. Zhang, R.-J., et al. *Spermatophyte Flora of Yinggeling Mountain, Hainan*. *Biodiversity Science*, 15(4): 382-392 (2007).
38. Plant Laboratory of Sun Yat-Sen University. *Plant Checklist of Bawangling National Reserve in Hainan*. (Scientific Report, 2004).
39. Tan, W.-F. *Biodiversity and Conservation System of Guangxi Shiwandashan Nature Reserve* (China Environment Science Press, 2005).
40. Zhao, N.-X., et al. *Integrated Study on Biodiversity of E'huangzhang Natural Reserve, Guangdong* (South China Botanical Garden, 2007).
41. Gao, Y.-D. *Floristic and Resources Study on the Seed Plants in Yunkaishan Nature Reserve* (Inner Mongolia Agricultural University, master dissertation, 2010).
42. Huang, Z.-L. *Comprehensive Investigation Report of Dinghushan National Nature Reserve in Guangdong Province* (Guangdong Science and Technology Press, 2015).
43. Wu, W.-Z., Chen, J.-H. & Wu, C.-C. *Scientific Survey of Guangdong Xiangtoushan Nature Reserve* (China Forestry Publishing House, 2003).
44. Plant laboratory of Sun Yat-sen University. *Plant Checklist of Heishiding Nature Reserve* (Sun Yat-Sen University, 1987).
45. Wu, L. *Studies on Species Diversity of Plants in Damingshan National Nature Reserve of Guangxi* (Guangxi Normal University, master dissertation, 2012).
46. Liao, W.-B. & Boufford, D. E. *A Checklist of the Vascular Plants of Taiwan* (Sun Yat-Sen University, manuscript and data, 2010).
47. Li, H.-T. *A study on Floristic Phytogeography of Seed Plants in Yuanjiang Nature Reserve, Yunnan* (Southwest Forestry College, master dissertation, 2008).
48. Chen, H.-F., Cui, X.-D. & Zhang, Y.-Y. *Plants of Nankunshan* (China Forestry Publishing

House, 2017).
49. Wang, J., Du, F., Yang, Y.-M., Tian, K. & Wang, Y.-X. *Scientific Survey and Research on Lancang River Nature Reserve in Yunnan Province, China* (Science Press, 2010).
50. Jiang, L.-Q. *The Preliminary Study on the Flora of Seed Plants in Yizu Mountain and their Use* (Yunnan University, master dissertation, 2018).
51. Xu, Y.-M. *Studies on Flora of Vascular Plant of Daguishan in Guangxi* (Guangxi Normal University, master dissertation, 2012).
52. Tan, W.-F. & Luo, B.-T. *Research and Conservation of Biodiversity in Guangxi Dayaoshan Natural Reserve* (China Environment Science Press, 2010).
53. Zhang, J.-Q. *Comprehensive Scientific Investigation of Shimentai National Nature Reserve in Guangdong Province* (Huazhong University of Science and Technology Press, 2017).
54. Liu, X.-Z., Xiao, Z.-Y. & Ma, J.-H. *Scientific Survey and Study on the Forest Ecosystem in Jiangxi Jiulianshan Nature Reserve* (China Forestry Publishing House, 2002).
55. Guangxi Institute of Botany. *Vascular Plants List of Xiling Mountain Nature Reserve*. (Guangxi Institute of Botany, 2015).
56. Xing, F.-W., Chen, H.-F., Wang, F.-G., Chen, Z.-M. & Zeng, Q.-W. *Catalogue of Plant Species Diversity in Nanling Mountains* (Huazhong University of Science and Technology Press, 2011).
57. Xiang, J.-F. *Study on the Flora of Seed Plants in Hunan Lanshan Forest Park* (Central South University of Forestry and Technology, master dissertation, 2009).
58. Ning, S.-J., Su, Y. & Tan, X.-F. *Critical Biodiversity Areas: Scientific Survey of the Jiuwanshan Nature Reserve in Guangxi* (Science Press, 2010).
59. Lin, P. *Comprehensive Scientific Investigation Report of the Liangyeshan Nature Reserve in Fujian* (Xiamen University Press, 2001).
60. Wei, S.-S. *Study on Flora of Vascular Plants and Rescource Protection Sanpihu Nature Reserve of Guangxi* (Guangxi University, master dissertation, 2017).
61. Ning, S.-J., Li, F. & He, C.-X. *Critical Biodiversity Areas: Scientific investigation on Yuanbao Mountain in Guangxi* (Guangxi Science and Technology Publishing House, 2009).
62. Yang, D.-D., Wu, Y.-M. & Yu, X.-L. *Biodiversity Research and Conservation of the Dupangling National Nature Reserve in Hunan* (Hunan Science and Technology Press, 2013).
63. Qiu, R.-W. *Study on the Flora of Seed Plants of Daguping Forestland in Shuangpai County, Hunan Province* (Central South University of Forestry and Technology, master dissertation, 2008).
64. Liu, X.-M., Guo, Y.-R. & Liu, R.-L. *The Collection of Scientific Exploration of Jiangxi Qiyunshan Nature Reserve* (China Forestry Publishing House, 2010).
65. Yi, R.-Y. *Study on the Flora of Seed Plants in the Bamian Mountain of Hunan Province* (Hunan Normal University, master dissertation, 2015).
66. Luo, Y. et al. *Scientific Investigation and Study of Yueliangshan Nature Reserve in Guizhou province* (China Forestry Publishing House, 2019).
67. Liu, R.-L., et al. *Comprehensive Scientific Survey of Ganjiangyuan National Nature Reserve in Jiangxi province* (China Forestry Publishing House, 2013).
68. Deng, X.-J., Jiang, Z.-G., Yang, Q.-L. & Li, X.-Y. *Comprehensive Scientific Investigation Report of the Jintongshan Nature Reserve in Hunan* (Hunan Normal University Press, 2011).
69. Guangxi Forestry Exploration and Design Institute. *Comprehensive Scientific Investigation Report of the Yinzhulaoshan Nature Reserve in Guangxi* (Scientific Report, 2013).
70. Wildlife Conservation Institute of Jiangxi Academy of Forestry & Jiangxi Suichuan Forestry Bureau. *Investigation Report of Nanfengmian Nature Reserve in Jiangxi* (Scientific Report, 2008).

71. Lin, P., Li, Z.-J. & Zhang, J. *Comprehensive Scientific Investigation Report of the Junzifeng Nature Reserve in Fujian* (Xiamen University Press, 2005).
72. Zhang, H.-H. & Zhang, X. *Biodiversity Study of Leigongshan National Nature Reserve* (Guizhou Science and Technology Publishing House, 2007).
73. Li, X. *Study on the Flora of Seed Plants in Dong'an Shunhuang Mountain National Nature Reserve of Hunan* (Central South University of Forestry and Technology, master dissertation, 2020).
74. Liao, W.-B., et al. *Study on Biodiversity of the Taoyuandong National Nature Reserve in Hunan Province* (Science Press, 2018).
75. Zhang, H.-H. *Scientific investigation Report of Nangong Nature Reserve* (Guizhou Science and Technology Publishing House, 2003).
76. Liao, W.-B., et al. *Integrated Study on Biodiversity of Mount Jinggangshan Regions In China* (Science Press, 2014).
77. He, L.-Z. & Liu, R.-L. *Scientific Survey and Biodiversity Study of the Qixiling Nature Reserve in Jiangxi Province* (Jiangxi Science and Technology Press, 2010).
78. Lin, P., Xie, D.-L. & Li, Z.-J. *Comprehensive Scientific Investigation Report of the Minjiangyuan Nature Reserve in Fujian* (Xiamen University Press, 2004).
79. Fujian Forestry Department. *Comprehensive Scientific Investigation Report of the Longxishan Nature Reserve* (Scientific Report, 1997).
80. Liu, B.-B. *The Floristic Geography of Spermatophyta in the North Section of Mt. Donggong* (Zhejiang A&F University, master dissertation, 2013).
81. Liu, X.-Z. & Fang, F.-S. *Comprehensive Scientific Investigation Report of the Gaotianyan Nature Reserve in Jiangxi*. (Scientific Report, 2007).
82. Chen G.-X., Zhang, D.-G. & Xiao, J.-W. *Inventory of Species Diversity of Vascular Plants in Wugongshan Areas* (Xin-an Jiaotong University Press, 2019).
83. Wang, B., Li, H.-J., Guo, Q.-S. & Xia, L.-F. *Research on Forest Biodiversity in Dagangshan Nature Reserve in Jiangxi* (China Forestry Publishing House, 2005).
84. Li, H. *Flora of Dulongjiang Region* (Yunnan Science and Technology Press, 1992).
85. Fujian Science and Technology Commission. *Scientific Investigation Report of the Wuyishan Nature Reserve* (Fujian Science and Technology Publishing House, 1993).
86. Liu, X.-Z. & Fu, Q. *Scientific Survey and Study of Rare Phytocoenosium on the Matoushan Nature Reserve in Jiangxi Province* (China Forestry Publishing House, 2006).
87. Guo, Y.-R., Jiang, B., Wang, Y.-Y., Yue, X.-G. & Ge, G. *Report of Scientific Survey on Mount Yangjifeng Reserve of Jiangxi* (Science Press, 2010).
88. Liu, X.-Z. & Fang, F.-S. *Scientific Survey of the Wuyishan Nature Reserve in Jiangxi* (China Forestry Publishing House, 2001).
89. Central South University of Forestry and Technology & Hunan Normal University. *Comprehensive scientific study of the Dawenshan Nature Reserve in Hunan* (Scientific Report, 2010).
90. Liu, X.-Z. & Wu, H.-P. *Scientific Survey and Study on the Guanshan Nature Reserve in Jiangxi Province* (China Forestry Publishing House, 2005).
91. Hunan Forestry Furvey Planning and Design Institute. *Comprehensive Scientific Investigation Report on Natural Resource of the Xiaoxi Nature Reserve in Hunan* (Scientific Report, 2000).
92. Peng, S.-L., et al. *Study on Biodiversity of Mount Sanqingshan in China* (Science Press, 2008).
93. Li, J.-X. *The Floristic Analysis of Seed Plants of Pingjiang Mufu Mountain in Hunan province* (Central South Forestry University, master dissertation, 2005).
94. Li, Z.-J., Wu, X.-P., Chen, X.-L. & Liu, C.-M. *Report of Scientific Survey on Jiulingshan*

Reserve of Jiangxi (Science Press, 2009).
95. Wildlife Conservation Institute of Jiangxi Forestry Science Academy & Jiangxi Wuning Forestry Bureau. *Comprehensive Scientific Investigation Report of Yishan Nature Reserve in Jiangxi* (Scientific Report, 2009).
96. Wang, Q.-F. & Ge, J.-W. *Investigation and Sonservation of Biodiversity of Jiugongshan Mountain Nature Reserve* (China Forestry Publishing House, 2002).
97. Liu, X.-Z. & Wang, L. *Scientific Survey and Study or Biodiversity on the Lushan Nature Reserve in Jiangxi province* (Science Press, 2010).
98. Deng, H.-P. *Plant Diversity of Jinyunshan National Nature Reserve in Chongqing* (Science Press, 2017).
99. Hu, Y.-M. & Gu, C.-M. *Research on the Biodiversity of Anhui Qingliangfeng National Nature Reserve* (Anhui Normal University Press, 2016).
100. Hu, J.-Q. & Liang, S.-W. *Plants of Huangshan Mountain* (Fudan University Press, 1996).
101. Ding, B.-Y., Li, G.-Y., Fu, C.-X. & Yang, S.-Z. *Flora of Tianmushan Mountain* (vol. 1-4) (Zhejiang University Press, 2010).
102. Zi, X.-Z. & Zhang, D.-C. *Flora of Dabieshan Mountain* (China Forestry Publishing House, 2006).
103. Deng, H.-P. *Biodiversity of Yintiaoling National Nature Reserve in Chongqing* (Science Press, 2018).
104. Zhang, Z.-J. *Research on Biodiversity in Tangjiahe National Nature Reserve, China* (Science Press, 2016).
105. Han, T. & Zheng, G.-H. *Scientific Investigation Report of Gaoleshan Nature Reserve in Tongbai, Henan Province* (Yellow River Water Conservancy Press, 2012).
106. Guo, W.-Y., Dang, K.-L. & Zhao, Y.-B. *Comprehensive Scientific Investigation and Study of Motianling Nature Reserve in Shaanxi Province* (Shaanxi Science and Technology Press, 2007).
107. Bai, Y.-X., Liu, J.-J. & Ma, X.-Q. *A Report of the Comprehensive Survey on Yuhe Nature Reserve in Gansu, China* (Gansu Science & Technology Press, 2012).
108. Yang, X.-Z., Liu, H. & Xu, T.-Q. *Biodiversity, Conservation and Management of Xinkailing Nature Reserve* (Shaanxi Science and Technology Press, 2012).
109. Li, Z.-G., Kang, K.-G. & Wu, Z.-H. *Comprehensive Scientific Investigation and Biodiversity Research in Pinglianghe Provincial Nature Reserve, Shaanxi Province* (Shaanxi Science and Technology Press, 2008).
110. Li, Z.-G., Ren, Y. & Wang, X.-J. *Comprehensive Investigation report of Changqing National Nature Reserve, Shaanxi province* (Shaanxi Science and Technology Press, 2016).
111. Ren, Y., Liu, M.-S., Tian, L.-H., Tian, X.-H. & Li, Z.-J. *Biodiversity, Conservation and Management of Taibaishan Nature Reserve* (China Forestry Publishing House, 2006).
112. Wang, X.-H. & Li, C.-R. *Biodiversity of Mount Taishan* (Intellectual Property Publishing House, 2013).
113. Zhao, J.-C., Wu, Y.-F. & Guan, W.-L. *Scientific Survey and Study on Biodiversity in Hebei Tuoliang Nature Reserve, China* (Science Press, 2008).
114. Liu, G.-Q., Wang, Z.-C. & Ma, G.-Q. *Beijing Yunmengshan Nature Reserve biodiversity* (Beijing Publishing House, 2006).
115. Chen, W., Qu, Z.-C. & Zhang, Y. *Biodiversity of Baishilizi National Nature Reserve in Liaoning province* (Liaoning Science and Technology Publishing House, 2017).
116. Wu, Y.-F., Zhao, J.-C. & Cheng, J. *Scientific Survey and Study on Biodiversity in Hebei Maojingba Nature Reserve, China* (Science Press, 2006).
117. Ding, T. *Studies on the Spermatiphytes Flora of Wangxia Limestone Area in Hainan Island*

(Sun Yat-sen University, master dissertation, 2002).
118. Wu, W.-H. *Studies on flora and phytogeography of Longgang National Nature Reserve of Guangxi, China* (Guangxi Normal University, master dissertation, 2011).
119. Yang, J.-C. *Study on Species Diversity of Plants in Longhushan Nature Reserve of Guangxi* (Guangxi University, master dissertation, 2013).
120. Tang, H. *Studies on the Flora of Vascular Plants of Laohutiao Nature Reserve of Guangxi* (Guangxi Normal University, master dissertation, 2010).
121. Zhang, H.-H., Long, Q.-D. & Liao, D.-P. *Comprehensive Scientific Investigation of Xingyi Pogang Nature Reserve* (Guizhou Science and Technology Publishing House, 2005).
122. Peng, R.-C. *Studies on Vascular Plants of Mulun National Natural Reserve, Guangxi, China* (Guangxi Normal University, master dissertation, 2013).
123. Luo, Y., et al. *Scientific Survey of the Wangmo Cycad Nature Reserve in Guizhou* (Guizhou Science and Technology Publishing House, 2010).
124. Scientific Research Department, Guizhou Maolan National Nature Reserve Administration. *Wild Animals and Plants Chenglist of the Maolan National Nature Reserve in Guizhou* (Scientific Report, 2014).
125. Zhu, J., et al. *Scientific Survey of Bada Mountain Nature Reseve in Panxian Country of Guizhou Province* (China Forestry Publishing House, 2013).
126. Zhang, H.-H., Zhou, Q. & Zhang, J.-G. *Comprehensive Scientific Survey of the Kuankuoshui Nature Reserve in Meitan* (Guizhou Science and Technology Publishing House, 2006).
127. Zhu, J., et al. *Scientific Survey of Siyetun Nature Reserve in Sinan County of Guizhou Province* (China Forestry Publishing House, 2017).
128. Yu, L.-F. *Scientific Survey of Kuankuoshui Nature Reserve* (Guizhou Science and Technology Publishing House, 2004).
129. Xiang, X.-M. *Flora of Seeds Plants in Dehang Geopark of Hunan Province* (Jishou University, master dissertation, 2021).
130. Gou, G.-Q., et al. *Biodiversity of Mayanghe National Nature Reserve in Guizhou* (Guizhou Science and Technology Publishing House, 2017).
131. Deng, H.-P. *Biodiversity of Jinfoshan National Nature Reserve in Chongqing* (Science Press, 2019).
132. Guizhou Forestry Department. *Background resources of Biodiversity in Dashahe Nature Reserve* (Guizhou Science and Technology Publishing House, 2006).
133. Xiao, Y. *Studies on Diversity of the Seed Plants in Wulongshan National Geopark* (Jishou University, master dissertation, 2015).
134. Liu, S,-X., Di, J.-P., Jiang, Y.-F., Gao, E.-H. & Lei, Y. *Hubei Qizimeishan Nature Reserve Scientific Survey and Research Report* (Hubei Science and Technology Press, 2006).
135. Ma, X.-Y. *A Floristic Study of the Vascular Plants on Jingshan Mountain Extension in Jingmen City, Hubei Province* (Wuhan Botanical Garden, the Chinese Academy of Sciences, master dissertation, 2006).
136. Xing, F.-W., et al. *Plant Resources and Protection of Shenzhen Qiniangshan Country Park* (China Forestry Publishing House, 2004).
137. Liu, H.-J., et al. *Comprehensive Scientific Survey of Biodiversity in Shenzhen Dapeng Peninsula Nature Reserve* (Sun Yat-Sen University Press, 2016).
138. Smith, S. A. & Brown, J. W. Constructing a broadly inclusive seed plant phylogeny. *Am. J. Bot.* 105, 302 – 314 (2018).
139. Jin, Y. & Qian, H. V.PhyloMaker2: An updated and enlarged R package that can generate very large phylogenies for vascular plants. *Plant Diversity* 44, 335 – 339 (2022).
140. Webb, C., Ackerly, D. & Kembel, S., Phylocom: Software for the Analysis of Phylogenetic

Community Structure and Character Evolution, with Phylomatic. version 4.2 https://phylodiversity.net/phylocom/ (2011).
