# Supplementary material for: Landform and lithospheric development contribute to the assembly of mountain floras in China
Source: Nat Commun. 2024 Jun 17;15:5139. doi: 10.1038/s41467-024-49522-4 (PMC11183111; doi:10.1038/s41467-024-49522-4)
Supplement: Supplementary file 4 — Reporting Summary [file 41467_2024_49522_MOESM4_ESM.pdf]

nature portfolio

Corresponding author(s): Qiang Fan, fanqiang@mail.sysu.edu.cn; Lei Wang, lwang@cnu.edu.cn; Su-Fang Chen, chsuf@mail.sysu.edu.cn; Wen-Bo Liao, lsslwb@mail.sysu.edu.cn

Last updated by author(s): Oct 19, 2022

Reporting Summary

Nature Portfolio wishes to improve the reproducibility of the work that we publish. This form provides structure for consistency and transparency in reporting. For further information on Nature Portfolio policies, see our Editorial Policies and the Editorial Policy Checklist.

Statistics

For all statistical analyses, confirm that the following items are present in the figure legend, table legend, main text, or Methods section.

n/a	Confirmed	
☐	☒	The exact sample size (n) for each experimental group/condition, given as a discrete number and unit of measurement
☒	☐	A statement on whether measurements were taken from distinct samples or whether the same sample was measured repeatedly
☐	☒	The statistical test(s) used AND whether they are one- or two-sided<br>*Only common tests should be described solely by name; describe more complex techniques in the Methods section.*
☐	☒	A description of all covariates tested
☐	☒	A description of any assumptions or corrections, such as tests of normality and adjustment for multiple comparisons
☐	☒	A full description of the statistical parameters including central tendency (e.g. means) or other basic estimates (e.g. regression coefficient) AND variation (e.g. standard deviation) or associated estimates of uncertainty (e.g. confidence intervals)
☐	☒	For null hypothesis testing, the test statistic (e.g. F, t, r) with confidence intervals, effect sizes, degrees of freedom and P value noted<br>*Give P values as exact values whenever suitable.*
☒	☐	For Bayesian analysis, information on the choice of priors and Markov chain Monte Carlo settings
☒	☐	For hierarchical and complex designs, identification of the appropriate level for tests and full reporting of outcomes
☐	☒	Estimates of effect sizes (e.g. Cohen's d, Pearson's r), indicating how they were calculated

Our web collection on statistics for biologists contains articles on many of the points above.

Software and code

Policy information about availability of computer code

Data collection	Mountain flora data was generation based on previously published flora data by manual input. The climate data of 140 mountain floras were extracted from CHELSA climate dataset at 30 arc-seconds resolution (v1.2, http://chelsa-climate.org/) based on geographical location of each mountain sites. Phylogenetic reconstruction was performed in R (http://www.r-project.org/) by the following package 'V.PhyloMaker2' . We also used the following packages in R4.1.0 to assist with data collection and data analyses: The Leipzig Catalogue of Vascular Plants (LCVP) lcvplants, ape (5.4-1), PhyloMeasures, picante and vegan (2.5-7). Landform type of each mountain was assigned based on the local scientific investigation report and World Geological Map (http://portal.onegeology.org/OnegeologyGlobal/).
Data analysis	Data analyses were performed in the R (version 4.1.0) and code is available at https://doi.org/10.5061/dryad.3n5tb2rkg

For manuscripts utilizing custom algorithms or software that are central to the research but not yet described in published literature, software must be made available to editors and reviewers. We strongly encourage code deposition in a community repository (e.g. GitHub). See the Nature Portfolio guidelines for submitting code & software for further information.

Data

Policy information about availability of data

All manuscripts must include a data availability statement. This statement should provide the following information, where applicable:

- Accession codes, unique identifiers, or web links for publicly available datasets
- A description of any restrictions on data availability
- For clinical datasets or third party data, please ensure that the statement adheres to our policy

Mountain floras dataset support the findings of this study are available within the previously published flora data included comprehensive species checklists, white papers, and research papers (Supplementary Table 1). The raw inventory floras data ('distribution_data_of_140_mountain_floras.csv') and dated phylogenetic tree are available at https://doi.org/10.5061/dryad.b2rbnzsk1. Data utilized data ('Inputdata data.csv') for analysis in this study are available in Source Data file.

Human research participants

Policy information about studies involving human research participants and Sex and Gender in Research.

Reporting on sex and gender	Not applicable
Population characteristics	Not applicable
Recruitment	Not applicable
Ethics oversight	Not applicable

Note that full information on the approval of the study protocol must also be provided in the manuscript.

Field-specific reporting

Please select the one below that is the best fit for your research. If you are not sure, read the appropriate sections before making your selection.

☐ Life sciences ☐ Behavioural & social sciences ☒ Ecological, evolutionary & environmental sciences

For a reference copy of the document with all sections, see nature.com/documents/nr-reporting-summary-flat.pdf

Ecological, evolutionary & environmental sciences study design

All studies must disclose on these points even when the disclosure is negative.

Study description	We tested how landform and bedrock development process affects the mountain flora species richness, flora age and flora phylogenetic structure. The result suggest the ages of mountain floras are consistent with the time when strata erosion, while species richness and floristic structure are closely related to the type of bedrock and degree of erosion. The main reason for landforms driving floristic assembly is that they represent or underlie major aspects of environmental filters to which plants respond via the processes of speciation, local extinction, and immigration from the regional species pool. Additionally, bedrocks, from which landforms are derived, may promote the evolution of endemic edaphic specialists, further driving differences in floras among landforms. We put forward 'floristic geo-lithology hypothesis'. Under this hypothesis, landforms develop according to their underlying bedrock, and their geological development drives both the assembly and subsequent differentiation of mountain floras. Our hypothesis provides a novel framework rooted in geology for future research on the origins, differentiation, and migration of angiosperm assemblages and mountain floras.
Research sample	We assembled a mountain flora dataset comprising 17,576 species of angiosperms from 140 well-studied mountain floras of China representing five landforms based on bedrock. This dataset contains more than half of the species in China and covers the major mountain landforms. This mountain floras includes 19 karst floras, 14 'karst-granitic' floras, 84 granitic floras, 13 desert floras, and 10 Danxia floras. Statistics show that 13005 species occurs in 84 granite mountains, 6941 species occurs in 19 karst landform mountains, 7423 species occurs in 14 'karst-granitic' mountains, 3146 species occurs in 10 Danxia landform mountains and 4077 species occurs in desert landforms mountains.
Sampling strategy	The 140 mountain flora could be divided into 5 mountain landforms based on their bedrocks. And the simple sizes of each kinds of landform are greater than or equal to 10 for statistical significance. We thus merged closely-ralated landforms into landform groups for these analyses.
Data collection	Liao W.-B., Zhao W.-Y., Liu Z.-C., Chen Z.-H., Wang Y., Chen J.-R., Xu K.-W., Shi S., Yin Q.-Y., Li X.-J. and D.E. Boufford compiled the mountain flora datasets. Zhao W.-Y. and Wang Y.-R. extracted the climate data for the 140 mountains. Zhao W.-Y., Liu Z.-C., Shi S., and Chen S.-F. calculated the flora phylogenetic diversity (PD.Faith), flora phylogenetic structure index (NRI, NTI, PDI), flora age (MDT, MDT.youngest, MDT.oldest) of each mountain.
Timing and spatial scale	The mountain floras data were conducted between 2009 to 2019, and represents the main mountain landforms in China.

Data exclusions	All the 140 mountain flora data were included in this study. This 140 mountain floras are well studied and representative. For the 19 climate variables, we excluded eight that had pairwise Pearson correlation coefficients > 0.95 to avoid collinearity.
Reproducibility	The process to obtain flora data and analysis method is clearly described in the Methods. Furthermore, the method of defining the types of mountain landforms is clearly.
Randomization	The 140 mountain flora is grouped by their bedrocks as described in the Methods. Because, current landforms and bedrocks consisted in a mountain is closely associated to the flora composition and development history in the mountain. The mountains with the same landform type have similar flora composition.
Blinding	Blinding was not relevant to our study, because no status was compared across sampled individuals.

Did the study involve field work? ☐ Yes ☒ No

Reporting for specific materials, systems and methods

We require information from authors about some types of materials, experimental systems and methods used in many studies. Here, indicate whether each material, system or method listed is relevant to your study. If you are not sure if a list item applies to your research, read the appropriate section before selecting a response.

Materials & experimental systems

n/a	Involved in the study
☒	☐ Antibodies
☒	☐ Eukaryotic cell lines
☒	☐ Palaeontology and archaeology
☒	☐ Animals and other organisms
☒	☐ Clinical data
☒	☐ Dual use research of concern

Methods

n/a	Involved in the study
☒	☐ ChIP-seq
☒	☐ Flow cytometry
☒	☐ MRI-based neuroimaging
